# Cannabidiol exerts antiinflammatory effects but maintains T effector memory cell differentiation in humans

**DOI:** 10.1172/jci.insight.198590

**Published:** 2025-11-18

**Authors:** Debora L. Gisch, Sachiko Koyama, Jumar Etkins, Gerald C. So, Daniel J. Fehrenbach, Jessica Bo Li Lu, Ying-Hua Cheng, Ricardo Melo Ferreira, Evan Rajadhyaksha, Kelsey McClara, Mahla Asghari, Asif A. Sharfuddin, Pierre C. Dagher, Laura M. Snell, Meena S. Madhur, Rafael B. Polidoro, Zeruesenay Desta, Michael T. Eadon

**Affiliations:** 1 Department of Medicine,; 2 Department of Pediatrics, and; 3 Department of Microbiology and Immunology, Indiana University School of Medicine, Indianapolis, Indiana, USA.

**Keywords:** Clinical Research, Immunology, Pharmacology, Transcriptomics, Transplantation

## Abstract

**BACKGROUND:**

Cannabidiol (CBD) is increasingly used for pain management, including in transplant recipients with limited analgesic options. Its immunomodulatory effects in humans are not well defined at a single-cell level at CBD steady state with concomitant tacrolimus treatment.

**METHODS:**

In a phase I ex vivo study, peripheral blood mononuclear cells from 23 participants who received oral CBD (Epidiolex) up to 5 mg/kg twice daily for 11 days were collected before CBD (pre-CBD) and at steady state (post-CBD). Lymphocytes were isolated and stimulated with anti-CD3/CD28 antibodies, with or without tacrolimus (5 ng/mL). Pharmacodynamic responses were assessed using CellTiter-Glo proliferation, single-cell and single-nucleus RNA sequencing, cytokine assays, and flow cytometry. Steady-state plasma concentrations of CBD were quantified via tandem mass spectrometry.

**RESULTS:**

We identified an increased proportion of T effector memory (TEM) cells post-CBD (22% increase), which correlated with CBD plasma concentrations (*R* = 0.77, *P* = 0.01). CBD reduced proliferation of T (37% decrease) and CD70^hi^ B (17% decrease) lymphocytes with additive immunosuppressive effects to tacrolimus. Single-cell RNA sequencing revealed reduced *IL2* and *TNF* signaling and altered receptor-ligand networks in TEM cells. Post-CBD cytokine assays revealed elevated proinflammatory IL-6 protein levels and antiinflammatory IL-10 levels, with reduced TNF-α, LTA, and IL-2. In flow cytometry, the proportion of TEM and TEMRA cells increased post-CBD with tacrolimus.

**CONCLUSION:**

CBD exerts mixed immunomodulatory effects in humans, combining antiproliferative and pro- and antiinflammatory responses. Understanding the clinical safety of CBD use is important given the paucity of pain control options available for immunocompromised transplant populations.

**TRIAL REGISTRATION:**

ClinicalTrials.gov NCT05490511

**FUNDING:**

NIH/National Center for Complementary and Integrative Health (R01AT011463); NIH/National Institute of General Medical Sciences (NIGMS) (R35GM145383); Intramural Research Program of the NIH; NIH/NIGMS (T32GM008425).

## Abstract 2

<span>We determine the effects of cannabidiol on lymphocyte populations in a clinical study of participants taking oral cannabidiol for 11 days with deep phenotyping: pharmacokinetic sampling, cell proliferation assays, cytokine measurement, flow cytometry, and scRNA sequencing of lymphocytes. </span>

## Introduction

Cannabidiol (CBD) is approved by the US Food and Drug Administration (FDA) for certain seizure disorders (1). CBD is also widely used off-label for the management of chronic pain (2). Individuals with chronic kidney disease or those who are transplant allograft recipients face challenges with analgesia because nonsteroidal antiinflammatory drugs are nephrotoxic and pose a significant risk for kidney function decline. CBD is a particularly attractive alternative for off-label pain control in these populations (3). However, special considerations are required for allograft recipients, who often receive calcineurin inhibitor–based (CNI-based) immunosuppressive regimens. Allograft recipients are at risk for pharmacokinetic drug-drug interactions (DDIs) with tacrolimus (TAC) and everolimus (4–6). However, less is understood regarding the interactions between CBD and CNIs within the immune system. This warrants particular attention because CBD and CNIs are involved in the deregulation of nuclear factor of activated T cells (NFAT) (7).

Allograft recipients require lifelong immunosuppression, placing them at risk for opportunistic infections (8). The FDA-approved form of CBD (Epidiolex) contains a warning regarding the increased risk of infection, without a mechanistic explanation for this adverse event (9). The immunomodulatory properties of CBD have been extensively studied in cell culture systems. For example, CBD has been shown to reduce T cell proliferation and proinflammatory cytokine secretion, in part through the suppression of NFAT and activator protein-1 (AP-1) (7, 10, 11). Most literature supports the antiinflammatory effect of CBD, but some studies also reveal a mixed picture of pro- and antiinflammatory effects (12). However, clinical studies addressing the impact of CBD on the immune system are lacking. Thus, we sought to understand the effects of CBD on the immune system in healthy participants before and after 2 weeks of CBD treatment.

In this study, we assessed the pharmacodynamic effects of CBD and DDI between CBD and tacrolimus on the immune system in human participants. Participants took 14 days of controlled Epidiolex administration at 5 mg/kg by mouth twice daily. An enriched set of their lymphocytes underwent single-cell RNA sequencing, proliferation assays, cytokine assessment, and flow cytometry before (pre-CBD) and after (post-CBD) 11 days of exposure to assess the comprehensive changes in the lymphocyte landscape. Pre-CBD and post-CBD blood samples were divided and sequenced with (CD3/CD28) and without (No CD3/CD28) anti-CD3/CD28 antibody stimulation to induce polyclonal T cell activation, and after stimulation with the addition of a defined concentration of tacrolimus (5 ng/mL) to assess the pharmacodynamic DDI (CD3/CD28 +TAC). We hypothesized that CBD would exert antiinflammatory effects and reduce immune cell proliferation. This hypothesis was only partially supported, as CBD led to a mixed set of pro- and antiinflammatory effects in the human participants. Some effects were synergistic, and others were antagonistic to tacrolimus-mediated ones. The immunomodulatory effects of CBD included significant concentration-dependent changes in T effector memory (TEM) cell proportions and cell-cell signaling.

## Results

### Participants.

**Figure 1:**
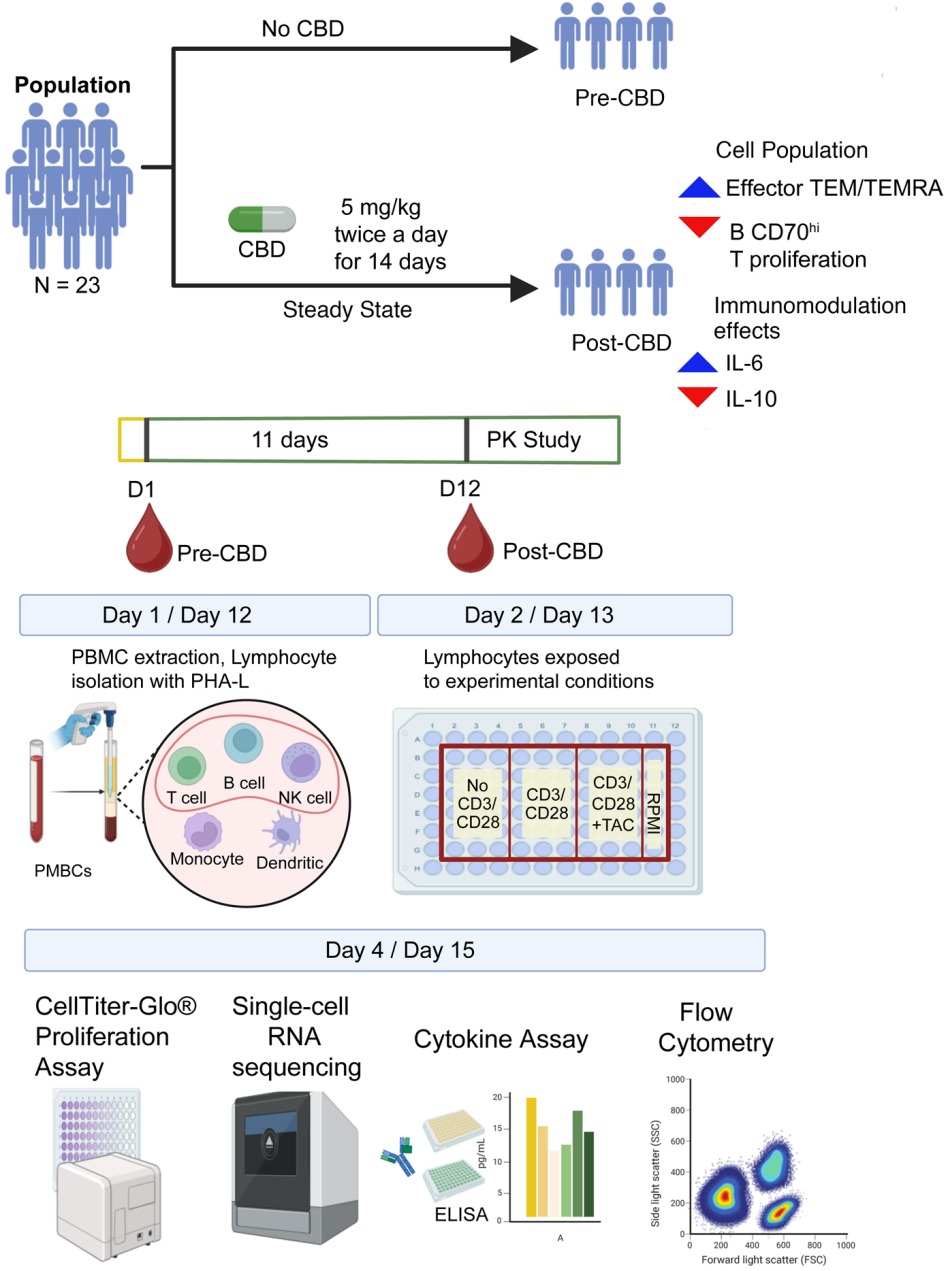
Operational workflow. The workflow is provided for the pharmacokinetic study of steady-state cannabidiol (CBD) in healthy human volunteers, highlighting the time points of sample acquisition for molecular analysis. Samples were acquired at 2 time points: pre-CBD, or before CBD administration (day 1); and post-CBD, at steady state (day 12). Each participant took oral CBD up to 5 mg/kg twice daily for 14 days. The blood samples underwent 72 hours of processing. On day 1/day 12, lymphocytes were isolated. On day 2/day 13, cells were divided into conditions: baseline without stimulation (No CD3/CD28), CD3/CD28 stimulation (CD3/CD28), and stimulated plus tacrolimus (CD3/CD28 +TAC). After 72 hours, cells were interrogated by CellTiter-Glo, scRNA-Seq, cytokine measurement, and flow cytometry. Between day 12 and day 14, participants underwent pharmacokinetic sampling for 48 hours to obtain CBD concentrations at steady state. Figure created in BioRender (Gisch D, 2025, https://BioRender.com/4la4844).

A total of 41 participants completed a pharmacokinetic study of CBD, which closed enrollment on July 15, 2025. Participants received 11 days of CBD at 5 mg/kg twice daily before steady-state pharmacokinetic sampling on day 12. A subset of participants (*N* = 23) provided blood samples for advanced phenotyping of their immune system before CBD exposure (pre-CBD) and at steady-state trough (post-CBD) just before the morning dose on day 12, to determine the effects of CBD on lymphocyte distribution, expression, and signaling. The peripheral blood samples underwent an experimental protocol to enrich the lymphocyte population (13). Advanced phenotyping included pre-CBD single-cell and single-nucleus RNA sequencing (sc/snRNA-Seq, *N* = 12), post-CBD scRNA-Seq (*N* = 10 with paired pre-CBD scRNA-Seq and pharmacokinetics), CellTiter-Glo proliferation assays (*N* = 19), cytokine assays (*N* = 3), and flow cytometry (*N* = 3). Supplemental Table 1 (supplemental material available online with this article; https://doi.org/10.1172/jci.insight.198590DS1) shows which assays were performed for each participant. A flow diagram (Figure 1) illustrates the study enrollment, timeline, and experimental technologies used to interrogate the specimens.

### The effects of CBD on immune cell proliferation and distribution.

**Figure 2:**
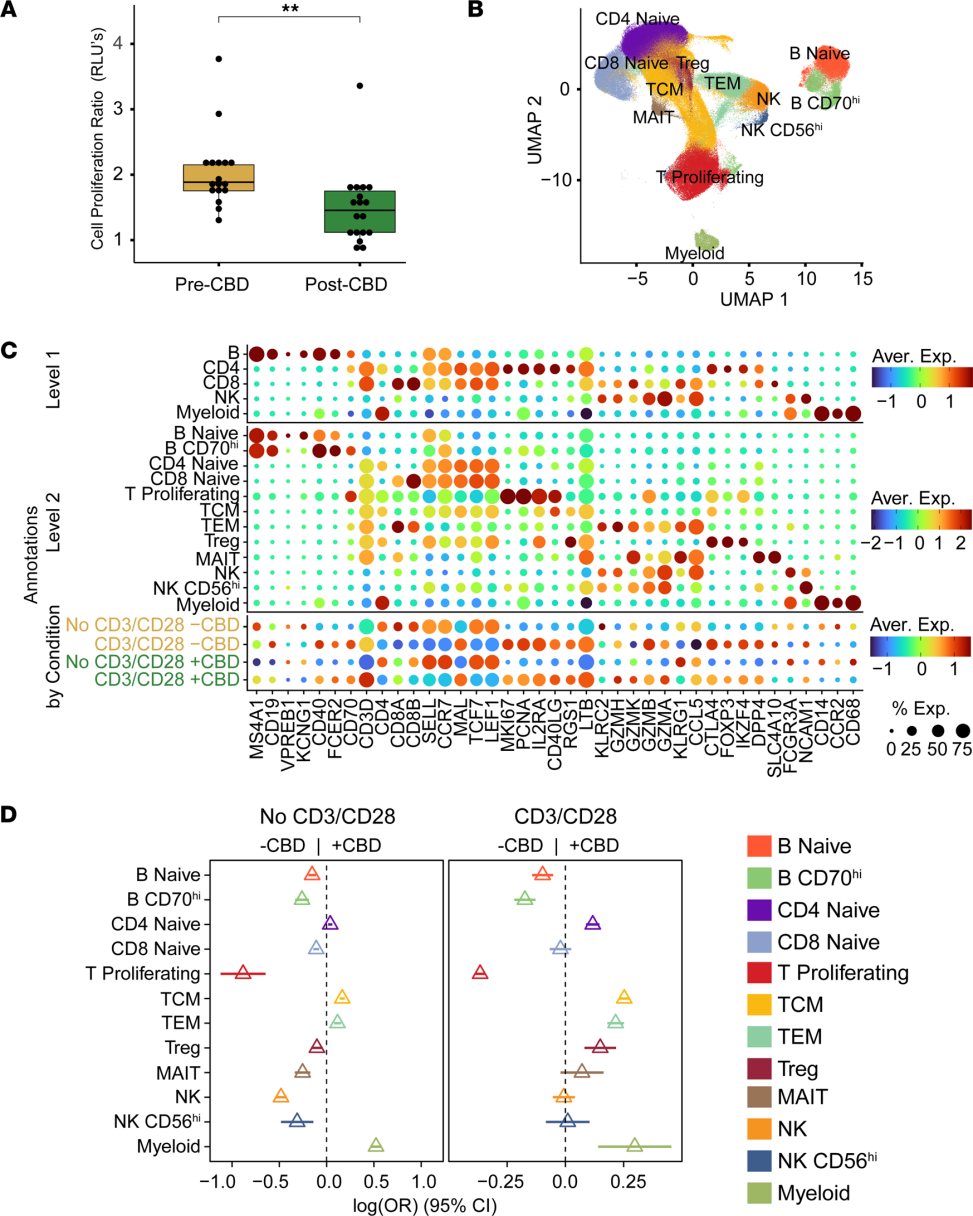
Molecular characterization of lymphocytes. Lymphocyte proliferation, annotation, and distribution were determined at pre- and post-CBD time points. (**A**) The CellTiter-Glo assay assessed cell viability at the pre-CBD (*N* = 17) and post-CBD (*N* = 18) time points. The proliferation ratio is the measured values of the stimulated (CD3/CD28) condition normalized to the No CD3/CD28 condition. Cell proliferation was reduced post-CBD (**P < 0.01 by 2-tailed *t* test). (**B**) UMAP with annotations from scRNA-Seq (*N* = 59) experiments at the pre-CBD and post-CBD time points for the CD3/CD28 and No CD3/CD28 conditions (321,327 cells displayed). Twelve cell type clusters were annotated: B naive, B CD70^hi^, CD4 naive, CD8 naive, T proliferating, T central memory (TCM), T effector memory (TEM), T regulatory (Treg), mucosal-associated invariant T (MAIT), natural killer (NK), natural killer CD56^+^ (NK CD56^hi^), and retained myeloid. (**C**) Canonical marker gene expression of PBMC types was used to annotate cell types. Three levels of annotation are provided: Level 1 includes B cells (MS4A1, CD19), CD4 lymphocytes (CD4), CD8 lymphocytes (CD8A, CD8B), NK cells (NCAM1), and myeloid cells (CD14, CCR2, and CD68). Level 2 annotations increased specificity with 12 cell types, including naive B, CD4 naive, CD8 naive, CD70^hi^, T proliferating, TCM, TEM, Treg, and MAIT. Dot size indicates the percentage of cells expressing each gene; color represents the average expression (log-normalized), from low (dark blue) to high (dark red). (**D**) Cell proportions changed between the pre-CBD and post-CBD time points in the No CD3/CD28 and CD3/CD28 conditions. An increase in TEM cells was observed post-CBD, while a decrease in B CD70^hi^ and proliferating T lymphocytes was observed. Forest plots display the log_2_ odds ratio between time points across the level 2 annotations. For each cell type, the triangle marks the estimated difference in proportions between pre- and post-CBD time points, and the horizontal line depicts its 95% CI.

Cell proliferation was assessed after anti-CD3/CD28 stimulation (CD3/CD28) at pre- and post-CBD time points using the CellTiter-Glo assay (Figure 2A). To assess proliferation, we calculated the ratio of luminescence in the CD3/CD28 condition to that in the No CD3/CD28 condition in both pre-CBD and post-CBD. Pre-CBD, the CD3/CD28 to No CD3/CD28 ratio was 2.04 ± 0.574. Post-CBD, the ratio was significantly reduced to 1.5 ± 0.571 (*P* = 0.0085), consistent with a 54% reduction in cell proliferation from individuals treated with CBD for 11 days (Supplemental Table 2). To understand the cellular heterogeneity underlying the differences observed in the cell proliferation assay, we performed scRNA-Seq on the No CD3/CD28 and CD3/CD28 conditions at the pre-CBD and post-CBD time points. After quality control, the scRNA-Seq dataset consisted of 314,746 cells across 4 conditions (Figure 2B): No CD3/CD28 –CBD (91,865 cells), No CD3/CD28 +CBD (54,914 cells), CD3/CD28 –CBD (108,423 cells), and CD3/CD28 +CBD (59,560 cells).

We clustered and annotated cells at both low (level 1) and high (level 2) resolution based on marker gene expression (Supplemental Table 3). The level 1 annotation consisted of 5 cell types (B, CD4, CD8, NK, and myeloid) used in downstream cross-cutting differential expression and pathway analyses. To assess granular cell type distribution, we used level 2 annotations to define 12 cell types (Figure 2C). These 12 subclusters represented the following cell types with parenthetical marker genes: naive B lymphocyte (*CD19*, *KCNG1*); B CD70^hi^ lymphocyte (*CD19*, *CD40*, *CD70*); naive CD4^+^ T lymphocyte (*CD4*, *SELL*, *CCR7*, *TCF7*); naive CD8^+^ T lymphocyte (*CD8B*, *SELL*, *CCR7*, *TCF7*); proliferating T lymphocyte (*CD3D*, *MKI67*, *PCNA*); T central memory (TCM) (*CD3D*, *SELL*, *RGS1*, *D40LG*); T effector memory (TEM) (*CD3D*, *KLRC2*, *GZMH*, *CCL5* with absence of *CCR7* and *SELL*); T regulatory lymphocyte (Treg) (*FOXP3*, *CTLA4*, and *IKZF4*); mucosal-associated invariant T (MAIT) (*DPP4*, *C4A10*); natural killer (NK) (*FCGR3A*); NK CD56^+^ or NK CD56^hi^ (*NCAM1*); and myeloid (*CD14*, *CCR2*, *CD68*). Only a limited population of myeloid cells were retained owing to our experimental selection for lymphocyte populations. Further subclustering of the proliferating T lymphocyte, TCM, and TEM clusters into CD4^+^ and CD8^+^ T lymphocytes was not feasible because they clustered based on their proliferative or memory features rather than CD4 and CD8 expression (Supplemental Figure 1). Within T cell subsets, TEM cells were enriched for CD8^+^ cells, whereas TCM cells were CD4 dominant; the proliferating T cell cluster contained both CD4^+^ and CD8^+^ cells with only minor double-negative and double-positive fractions (Supplemental Figure 1D).

The immune cell distribution of participants changed significantly after 11 days of CBD administration (Figure 2D and Supplemental Table 4). In the No CD3/CD28 baseline condition, the proportion of proliferating T and B lymphocytes, naive B lymphocytes, MAITs, and NK cells was reduced post-CBD compared with pre-CBD. Although the absolute quantity of all cell types was reduced post-CBD, we observed significant relative increases in TCM and TEM cells when comparing the same conditions. With CD3/CD28 stimulation, we again observed reductions in proliferating T lymphocytes (36% reduction, 95% CI: –39 to –34), B CD70^hi^ lymphocytes (17% reduction, 95% CI: –22 to –13), and naive B lymphocytes (10% reduction, 95% CI: –14 to –5) post-CBD. A greater proportion of TCM cells (25% increase, 95% CI: 23 to 27) and TEM cells (22% increase, 95% CI: 18 to 25) were also observed post-CBD. One key difference between the CD3/CD28 and No CD3/CD28 conditions was the change in Treg proportion, which increased post-CBD with CD3/CD28. Myeloid cell populations were upregulated post-CBD, but we interpret these results with caution given our experimental design. We also examined B cell subsets and found a minor increase in B memory cells after CD3/CD28, but no consistent upregulation of B memory or plasma cell populations across conditions, and the magnitude of change was smaller than that observed for TEM cells.

Finally, we identified changes in expression patterns across all level 1 and level 2 resolution cell types in the CD3/CD28 and No CD3/CD28 conditions between participants pre-CBD and post-CBD (Supplemental Figure 2 and Supplemental Table 5). *CD69*, an early activation receptor, was downregulated in naive T cells post-CBD. *KIT*, a type III receptor tyrosine kinase involved in hematopoiesis, was downregulated in proliferative T cells post-CBD, but was not differentially expressed in the Treg population.

### CBD increases TEM cell proportion in a concentration-dependent manner.

**Figure 3:**
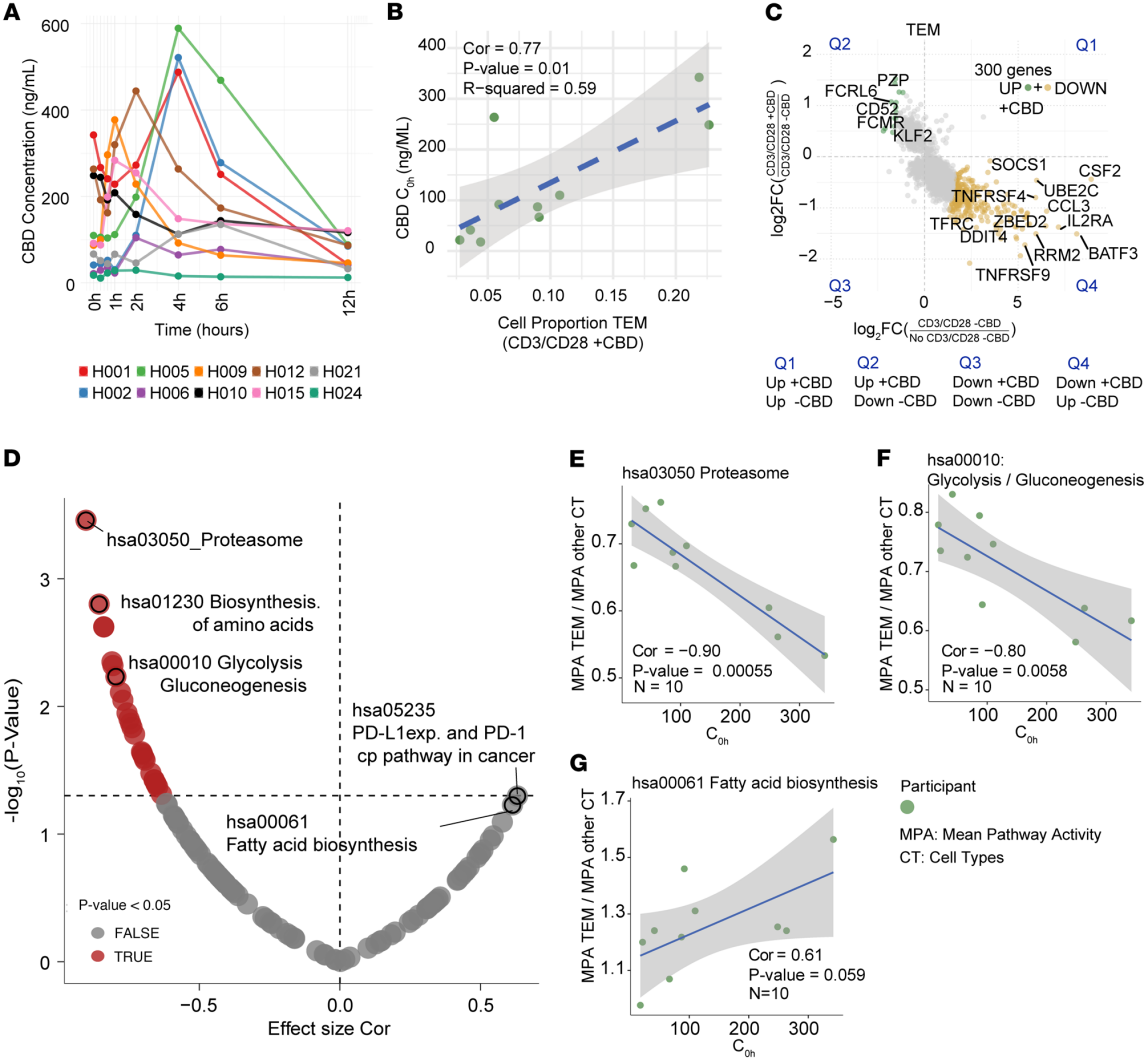
CBD concentration–dependent effects on cell proportion. In the post-CBD phase, 10 participants had overlapping sc/snRNA-Seq and pharmacokinetic data. (**A**) Concentration-to-time curves of CBD concentration levels (ng/mL) at time points starting on day 12: 0 hours, 0.33 hours, 0.66 hours, 1 hour, 2 hours, 4 hours, 6 hours, and 12 hours while at CBD steady state. Some inter-individual variability was observed in the pharmacokinetic profiles of participants. (**B**) Scatterplot shows relationships between immune cell proportions and CBD concentrations in participants. Green dots represent proportion of TEM cells versus CBD trough concentration at steady state (C_0h_), with Pearson’s correlation coefficient (Cor) of 77%, *P* value of 0.01, and *R*^2^ value of 0.59. Dashed line indicates linear regression fits, performed using Pearson’s correlation by lm method in ggplot2 (v3.5.1). (**C**) Gene expression in TEM cells changed across comparisons: No CD3/CD28 –CBD vs. CD3/CD28 –CBD (*x* axis) and CD3/CD28 +CBD vs. CD3/CD28 –CBD (*y* axis). Scatterplot depicts log_2_ fold changes (log_2_FC) in gene expression for TEM cells in each comparison. Each point represents a gene with statistically significant differential expression (adjusted *P* value < 0.05) in both comparisons. Genes exhibiting opposing directions of regulation — up in one comparison and down in the other — are highlighted (green, upregulated with CBD under stimulation; gold, downregulated with CBD under stimulation). The top 300 genes identified based on discordant regulation across CBD treatment conditions and ranked by Euclidean distance from the origin (see Supplemental Table 6) were analyzed using active-subnetwork enrichment via pathfindR. Genes with adjusted *P* value less than 0.05 were input into pathfindR using the BioGRID protein-protein interaction network and KEGG gene set. (**D**) Volcano plot showing associations between CBD trough concentration (C_0h_) and pathway activity in TEM cells. Each point represents a pathway, with the *x* axis indicating effect size (correlation coefficient) and the *y* axis showing statistical significance (–log_10_ *P* value). *N* = 10 participants. (**E**–**G**) Negative correlations were observed for KEGG hsa03050 (proteasome, 90%, *P* = 0.00055; Pearson’s) and hsa00010 (glycolysis/gluconeogenesis, 80%, *P* = 0.0058; Pearson’s), while a positive correlation was identified for hsa00061 (fatty acid biosynthesis, 61%, *P* = 0.059; Pearson’s). For each participant in the *x* axis CBD trough C_0h_ and *y* axis mean pathway activity (MPA) TEM cell divided by MPA calculated for all other cell types (CT).

A group of participants (*N* = 10) had both pharmacokinetic and scRNA-Seq data post-CBD (Table 1). The study population had a median age of 42 ± 18.5 years, and 80% were female. Most individuals self-identified as White or European American (70%). Plasma concentrations of CBD were assessed for the steady-state phase of CBD administration on day 12 at 8 sampling time points over a 12-hour dosing interval (Figure 3A). Pharmacokinetic parameters were derived by noncompartmental analysis (Table 2). The mean CBD maximal concentration at steady state (C_max,ss_) was 438.0 ng/mL (95% CI: 263.5 to 612.5 ng/mL). The mean trough concentration at steady state (C_0h,ss_) was 141.5 ng/mL, with substantial variability of 95% CI 64.2 to 218.8 ng/mL. Because we had identified an increase in TEM cells post-CBD, we tested whether the TEM proportion correlated with either C_0h,ss_ or C_max,ss_. The strongest correlation between concentration and TEM proportion was seen at the C_0h,ss_ time point (correlation *R* = 0.77, *P* = 0.01), when the scRNA-Seq data were acquired (Figure 3B). We identified a trend toward a negative correlation between proliferative T cells and increasing CBD concentrations at multiple time points. Because the absolute quantities of all cell types were reduced post-CBD, the increased TEM proportion is due to a relatively greater reduction in non-TEM cell abundance post-CBD. We did not identify a significant positive or negative correlation with the Treg proportion. We next made 2 comparisons of differentially expressed genes (DEGs) to filter the genes most affected by CBD: (a) CD3/CD28 –CBD versus No CD3/CD28 –CBD to remove genes most affected by stimulation and experimental conditions, and (b) CD3/CD28 –CBD versus CD3/CD28 +CBD to then identify genes specifically related to CBD administration regarding T cell receptor stimulation (Figure 3C). Genes in quadrant Q2 were upregulated in the CD3/CD28 +CBD condition. Still, they were downregulated in the No CD3/CD28 –CBD condition, representing an enhanced response in the CD3/CD28 CBD condition relative to both the No CD3/CD28 –CBD and CD3/CD28 –CBD. Conversely, genes in quadrant Q4 were downregulated in CD3/CD28 CBD and upregulated in CD3/CD28 –CBD, indicating a suppressed response in the CD3/CD28 +CBD condition. This quadrant-based pattern highlights distinct regulatory shifts driven by the CBD (Supplemental Table 6). We selected the top 300 genes farthest from the origin for Kyoto Encyclopedia of Genes and Genomes (KEGG) pathway enrichment analysis to identify 112 significantly enriched pathways (Supplemental Table 7). The pathway analysis concentration showed that for TEM cells, the computed participant-level average pathway activity scores were compared as well as their ratios (TEM vs. all other cells). Correlations between baseline concentration (C_0h_) and pathway ratios were evaluated using Pearson’s correlation test (Figure 3D). Among the pathways, we identified a negative correlation with “proteasome” 90% enrichment and increasing CBD concentration (Figure 3E). Low proteasome activity has been associated with differentiation to effector cell state (14). We also identified a negative correlation between glycolysis and increasing CBD concentration (Figure 3F) and a positive correlation with fatty acid biosynthesis (Figure 3G), consistent with a shift from activated CD8^+^ T cell to long-term memory function as has been previously described (14).

### CBD modulates intercellular communication and pathways of TEM cells.

**Figure 4:**
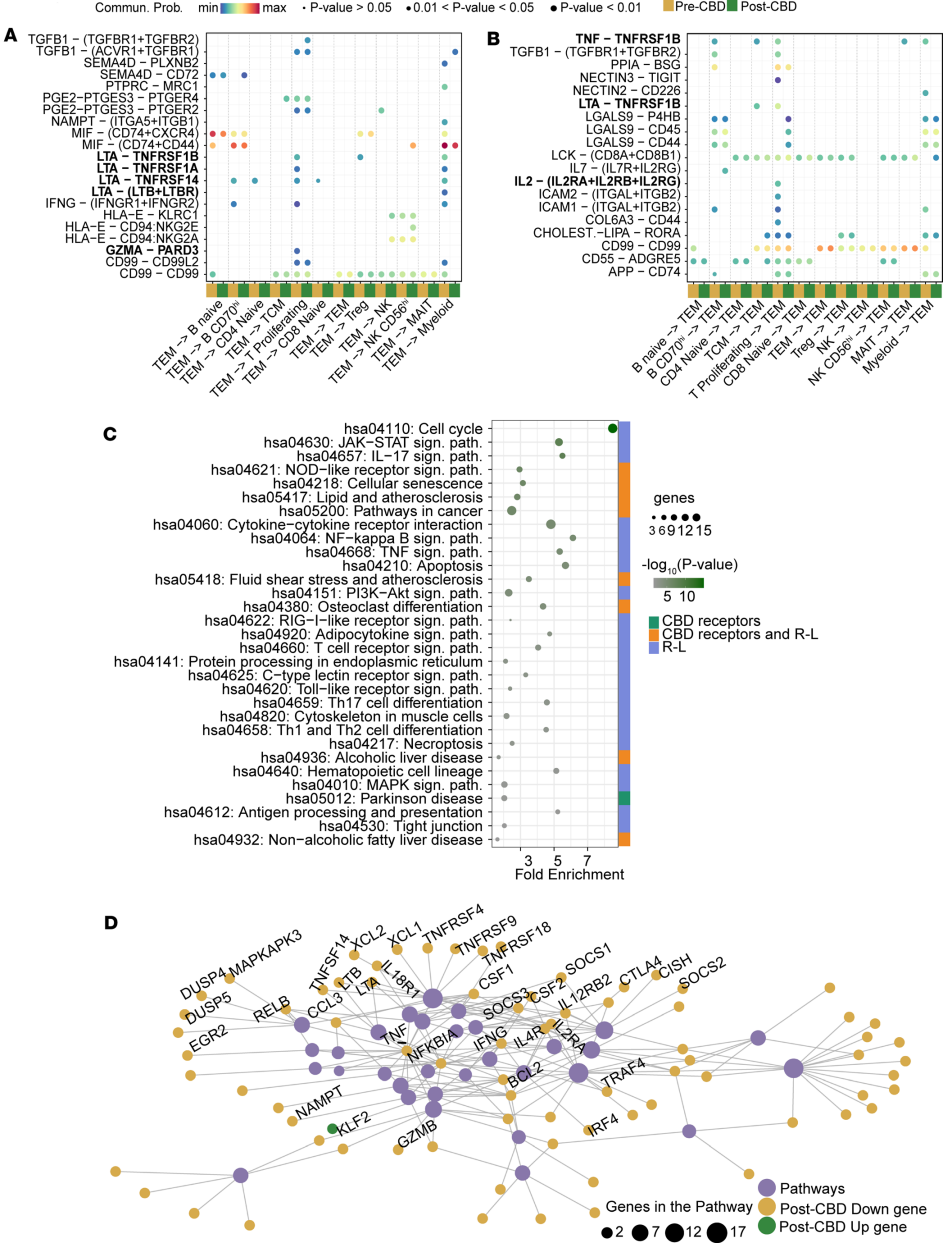
Receptor-ligand interactions and expression signature of TEM cells. Receptor-ligand (R-L) interactions, expression signature, and pathways of TEM cells were explored pre-CBD and post-CBD. (**A**) Outgoing signals from TEM cells (source) to all other immune cell types (targets) are displayed in the dot plot, both pre-CBD (dark yellow) and post-CBD (dark green). Directional ligand-receptor (L-R) signaling interactions involving TEM cells change before and after CBD titration. Communication was lost from TEM cells to B CD70^hi^ lymphocytes, proliferating T lymphocytes, and naive CD4^+^ cells through the LTA-TNFRSF14 R-L, LTA-TNFRSF1B, and LTA-TNFRSF1A R-L signals. (**B**) Incoming signals to TEM cells (target) from all other cell types (sources) are displayed. LTA-TNFRSF1B signaling was lost in proliferating T lymphocytes and TCM cells because of a loss of TNFRSF1B expression in TEM cells. Signaling through TNF to TNFRSF1B was lost in B CD70^hi^, proliferating T, and TCM lymphocytes. IL-2 signaling through IL-2 receptors was lost between proliferative T lymphocytes and TEM cells. (**A** and **B**) Communication probability (Commun. Prob.) was compared between pre- and post-CBD conditions. Bubble size represents significance of the L-R pair (*P* value, permutation test). Communication probability is indicated by colormap maxima (red) and minima (blue). Axis labels indicate interacting cell types, with TEM cells consistently involved as a source or target in each panel. The set R-L on the plot was filtered to display R-L pairs activated or inhibited by the CBD condition. (**C**) Enriched pathways were subsequently filtered to retain only those containing known CBD receptors or R-L interactions, prioritizing biologically relevant mechanistic processes. The enrichment chart displays the filtered pathways, ranked by –log_10_(*P*), representing those most plausibly linked to CBD’s immunomodulatory actions in the context of TEM cell regulation. (**D**) Graph-based network visualization of enriched KEGG pathways from TEM cells displays the pathways (purple), adding the DEGs on the pathways (post-CBD). *KLF2* was the only upregulated gene.

To investigate the mechanism of how TEM cells respond to CBD, we profiled cell-cell communication comparing pre-CBD and post-CBD receptor-ligand (R-L) pairs. With TEM as the source, we identified 9 R-L pairs turned on post-CBD and 13 R-L pairs turned off post-CBD (Figure 4A). Lymphotoxin-α (LTA) or tumor necrosis factor-β (TNF-β) signaling was altered in multiple cell types. For example, communication was lost from TEM cells to B CD70^hi^ lymphocytes, proliferating T lymphocytes, naive CD4^+^ cells, and myeloid cells through the R-L pair LTA-TNFRSF14. Signaling through the R-L pairs LTA-TNFRSF1B and LTA-TNFRSF1A was also lost in proliferating T lymphocytes.

In the reverse direction, with TEM as the target cell, we found 8 R-L pairs turned on post-CBD and 11 R-L pairs turned off post-CBD (Figure 4B). LTA-TNFRSF1B signaling was again lost in proliferating T lymphocytes, and now in TCM cells, because of a loss of *TNFRSF1B* expression in TEM cells. Analogously, signaling through TNF to TNFRSF1B was lost in B CD70^hi^, T, and TCM lymphocytes. Interleukin-2 (IL-2) signaling through IL-2 receptors was also disrupted between the proliferative T lymphocytes and the TEM cells. Post-CBD, increased signaling between the Tregs and TEM cells was predicted through LCK–(CD8A+CD8B1) and multiple HLA proteins with either CD8A or CD8B. The complete list of R-L changes between pre-CBD and post-CBD for all 12 cell types is available in Supplemental Table 8.

We then applied an additional literature filter (3) to prioritize pathways that include known receptors targeted by CBD or genes/proteins involved in R-L interactions previously identified in the cell-cell communication analyses for TEM cells. This integrated approach highlighted a subset of biologically relevant pathways (*N* = 72 with subset in Figure 4C). The pathways included cell cycle arrest, JAK/STAT signaling, IL-17 signaling, NOD-like receptor signaling, and cellular senescence, all representing key mechanisms through which CBD modulates the immune cell environment (3). Among the genes annotated within these pathways in the KEGG database, we identified several previously described CBD-interacting receptors, including *PPARG*, a transcription factor, present in the osteoclast differentiation, lipid, atherosclerosis, and non-alcoholic fatty liver disease pathways (15). The cation channel TRPV2 is also a known interacting protein for CBD and is present in the NOD-like receptor signaling, TRPV4, fluid shear stress, atherosclerosis, and cellular senescence pathways (16, 17). *TNF* and *IL2RA* were both downregulated in TEM cells after CBD administration, and members of over 50 pathways were enriched in the post-CBD condition. Among DEGs, most were downregulated in the post-CBD condition (Figure 4D); however, one of the upregulated genes was *KLF2*, a transcription factor with a purported role in CD8^+^ T cell differentiation and suppression of exhaustion (Supplemental Table 9) (18–20). *KLF2^+^* T cells produce IL-10, may be a subpopulation of T regulatory type 1 cells, and possess an antiinflammatory phenotype (21).

### Proliferation of B and T lymphocytes.

**Figure 5:**
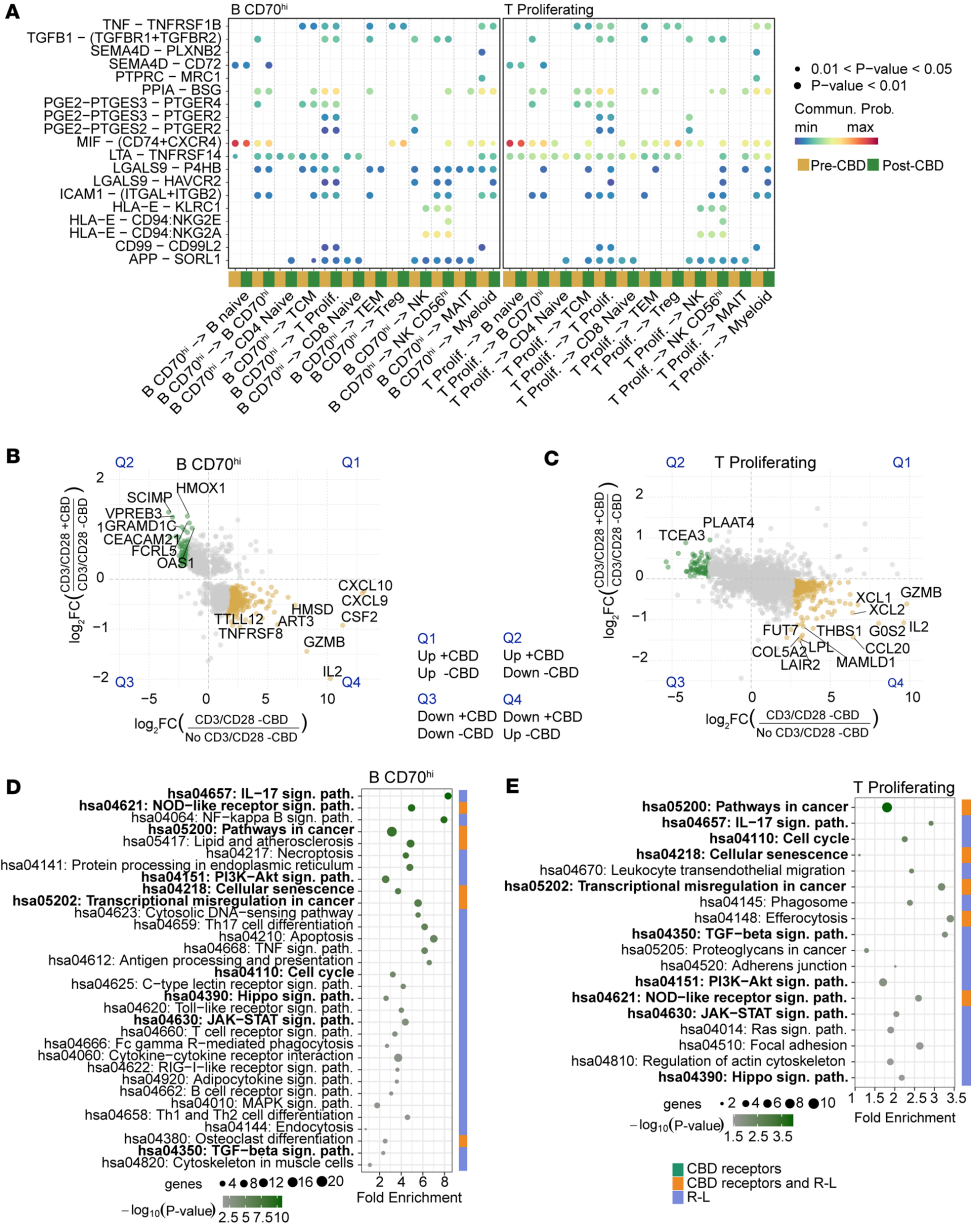
B CD70^hi^ and proliferating T cell types. The R-L interactions, expression signature, and pathways of B CD70^hi^ and proliferating T cells were assessed pre-CBD and post-CBD. (**A**) B CD70^hi^ and proliferating T cells were analyzed as the source population sending signals to other immune cells and themselves. R-L interactions that changed post-CBD included TNF-TNFRSF1B, TGFB1–(TGFBR1+TGFBR2), and ICAM1–(ITGAL+ITGB2). R-L communication profiles were inferred using CellChat for cells pre- and post-CBD treatment. Bubble plots show directional ligand-receptor (L-R) signaling where post-CBD has inhibition or activation. (**B** and **C**) Gene expression patterns in B CD70^hi^ (**B**) and T (**C**) cells exhibited similarities across 2 comparisons: No CD3/CD28 –CBD vs. CD3/CD28 –CBD (*x* axis) and CD3/CD28 +CBD vs. CD3/CD28 –CBD (*y* axis). Scatterplots depict the log_2_ fold changes (log_2_FC) in gene expression. Each point represents a gene with statistically significant differential expression (adjusted *P* value < 0.05) in both comparisons. Genes exhibiting opposing directions of regulation, up in one comparison and down in the other, are highlighted (green, upregulated with CBD under stimulation; gold, downregulated with CBD under stimulation). (**D** and **E**) The top 300 genes were ranked by Euclidean distance from the origin and used for pathway analyses for B CD70^hi^ cells (**D**) and T cells (**E**) via pathfindR. Genes with FDR-adjusted *P* value less than 0.05 were input into pathfindR using the BioGRID protein-protein interaction network and KEGG gene set. Enriched pathways were subsequently filtered to retain only those containing known CBD receptors or R-L interactions. The enrichment chart displays the filtered pathways, ranked by –log_10_(*P* value), representing those most plausibly linked to CBD’s immunomodulatory actions.

Cellular proliferation in response to CD3/CD28 was reduced post-CBD, as evidenced by the proliferation assay (Figure 2A) and scRNA-Seq (Figure 2D). To explore the mechanism underlying this reduction, we examined cell-cell communication through R-L pairs in B CD70^hi^ and proliferating T lymphocytes (Figure 5A). Three R-L interactions were changed post-CBD: TNF-TNFRSF1B, TGFB1–(TGFBR1+TGFBR2), and ICAM1–(ITGAL+ITGB2). The last R-L pair was turned on (i.e., opposite direction of effect), with TCM cells increasing *ITGAL*+*ITGB2* ligand expression post-CBD (Supplemental Table 8). We again assessed the top 300 DEGs to examine B CD70^hi^ and proliferating T lymphocyte pathways (Figure 5, B and C, and Supplemental Tables 10–13). Using an analysis parallel to the TEM cell analysis, we filtered pathways by known CBD receptors and R-L pairs. B CD70^hi^ and proliferating T lymphocyte–enriched pathways revealed strong overlaps, including IL-17 signaling, cell cycle, TGF-β signaling, and JAK/STAT signaling. The sets of genes that overlapped and had the same direction of effect in both cell types were *CCND2*, *HSP90AB1*, *IL2*, *ID2*, *NAMPT*, *DHX33*, *TRAF4*, *HSPD1*, *KSR1*, and *GZMB*, all downregulated, and *RASGRP2* and *CCND2*, which were upregulated. These DEGs were present in multiple overlapping pathways (Figure 5, D and E). Overall, these data support that CBD treatment retards T cell proliferation through reductions in cell-cell signaling (TNF, TGFBR1, and IL-2).

B lymphocytes are known to express cannabinoid receptor 2 (CB2, *CNR2*) on their cell surface, and reduced CB2 expression is associated with reduced cell proliferation (22). Commensurate with this observation, we found greater expression of *CNR2* in B CD70^hi^ cells than in naive B cells (Supplemental Figure 3). Further, the expression of *CNR2* was reduced post-CBD in all conditions, again consistent with CBD’s antiproliferative effects.

### Pharmacodynamic interactions of CBD and tacrolimus.

**Figure 6:**
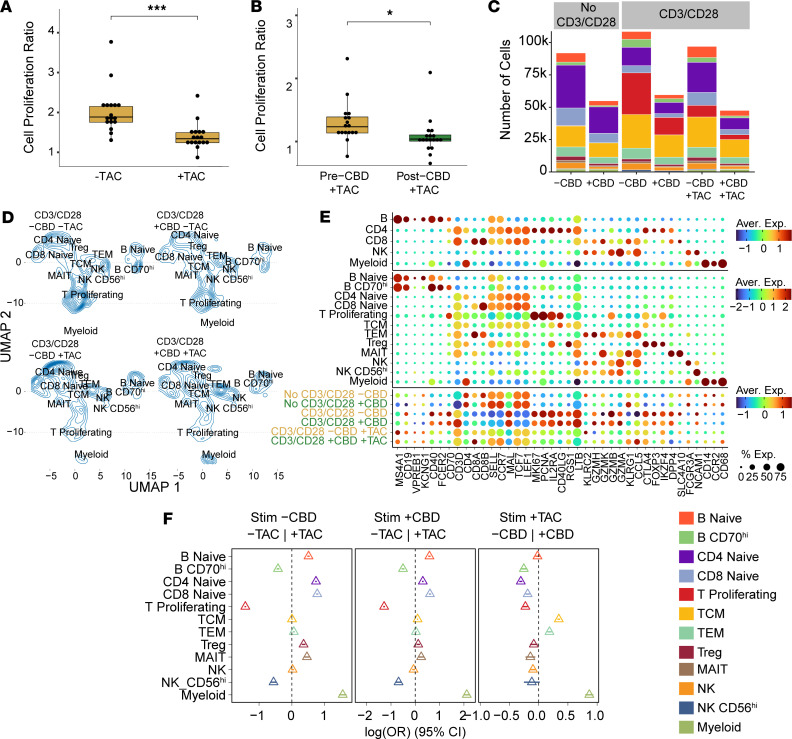
Pharmacodynamic interactions between CBD and tacrolimus. Immune interactions between CBD and tacrolimus (TAC) were explored. Cells were exposed to CBD in vivo for 11 days, whereas TAC was added to cells ex vivo. (**A**) Cell proliferation was assessed by CellTiter-Glo assay in 2 conditions: CD3/CD28 –TAC (*N* = 17) and CD3/CD28 +TAC (*N* = 17). As expected, CD3/CD28 +TAC had reduced proliferation in the absence of CBD compared with CD3/CD28 –TAC (***P < 0.001, 2-tailed *t* test with Bonferroni’s correction for multiple comparisons). (**B**) The effect of TAC on proliferation with and without CBD was compared in the pre-CBD (*N* = 17) and post-CBD (*N* = 18) time points. The relative proliferation of post-CBD +TAC was reduced compared with the pre-CBD +TAC condition (*P < 0.05, 2-tailed *t* test). All CD3/CD28 conditions were normalized to the No CD3/CD28 conditions to determine change in proliferation in response to CD3/CD28. (**C**) Quantitation of absolute cell number in each scRNA-Seq condition with/without CBD and TAC, split by level 2 annotation. Number of cells: No CD3/CD28 –CBD –TAC = 91,865; No CD3/CD28 +CBD –TAC = 54,914; CD3/CD28 –CBD –TAC = 108,423; CD3/CD28 +CBD –TAC = 59,560; CD3/CD28 –CBD +TAC = 96,914; CD3/CD28 +CBD +TAC = 47,468. (**D**) scRNA-Seq cell density plot across conditions with/without CBD and TAC in the stimulated conditions. (**E**) Marker gene expression comparisons at different annotation resolutions and across the 6 conditions. Dot plot shows average expression and cell percentage of pan-marker genes for PBMCs and B, T, NK, and myeloid cells. (**F**) Cell type distribution is expressed as an odds ratio for the level 2 annotation and displayed in a forest plot for 3 comparisons: left, pre-CBD *–*TAC vs. pre-CBD +TAC; middle, CD3/CD28 post-CBD *–*TAC vs. CD3/CD28 post-CBD +TAC; right, CD3/CD28 pre-CBD +TAC vs. CD3/CD28 post-CBD +TAC. For each cell type, the triangle marks the estimated difference in proportions between pre- and post-CBD time points, and the horizontal line depicts its 95% CI. A CI that intersects the vertical dashed null line indicates no statistically significant difference, whereas intervals that remain entirely on one side denote significance at *P* < 0.05.

The pharmacokinetic interaction between tacrolimus and CBD has been reported (5). We sought to determine whether a pharmacodynamic interaction was also present between these drugs. Study participants took 11 days of oral CBD; in contrast, tacrolimus (5 ng/mL) was administered ex vivo to the CD3/CD28–stimulated lymphocytes of each participant (for 48 hours) to avoid concentration variability resulting from the known pharmacokinetic interaction in vivo. As a positive control, we confirmed that tacrolimus reduced cell proliferation (*P* < 0.0001; Figure 6A). We found that the reduction in proliferation was additive to that of CBD (*P* < 0.01; Figure 6B). We expanded the scRNA-Seq object to 459,144 cells by adding the CD3/CD28–stimulated plus tacrolimus conditions pre- and post-CBD. The absolute distribution of cells varied across the 6 conditions (No CD3/CD28, CD3/CD28, and CD3/CD28 with tacrolimus, each pre- and post-CBD; Figure 6C). As expected, B CD70^hi^ and proliferating T lymphocytes decreased in the tacrolimus conditions (Figure 6D). Although there were substantial differences in cell type distribution, the expression patterns and cell cluster annotations were similar after tacrolimus addition (Figure 6E and Supplemental Table 14). As expected, tacrolimus administration proportionally reduced lymphocyte proliferation, with a relative increase in naive B and T lymphocytes, both pre- and post-CBD (Figure 6F). The effects of CBD on TEM and TCM proportions were maintained even after tacrolimus addition (Figure 6F and Supplemental Table 15). Across all 6 conditions, we annotated unsupervised DEGs, supervised marker genes, CBD receptor expression, and cytokine expression. Genes associated with proliferation (*DLGAP5*, *KIF18B*, *KIF20A*, *MKI67*, *PBK*) were upregulated after CD3/CD28, but downregulated post-CBD (Supplemental Figure 3). The expression of *FABP5*, a purported CBD receptor, was decreased in the post-CBD conditions and with tacrolimus. Likewise, tacrolimus and CBD both prevented upregulation of transcript expression of the cytokines *CSF2*, *IL2*, *LTA*, and *TNF* (Supplemental Figure 3).

### Cytokine protein response to CBD.

**Figure 7:**
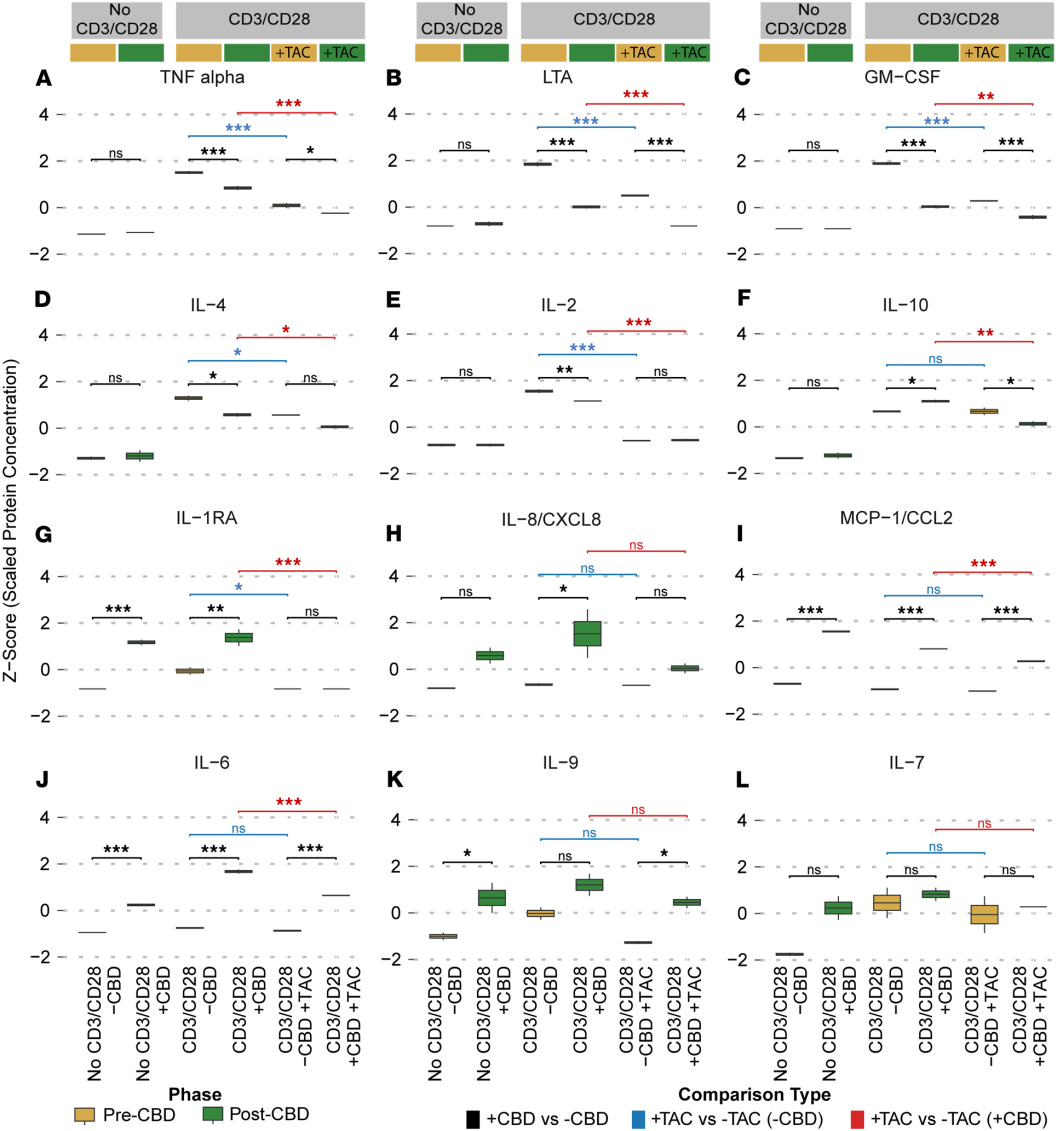
Cytokine levels. The effects of CBD treatment and/or tacrolimus (TAC) exposure on cytokine and chemokine levels were assessed under different conditions. CBD was administered in vivo over 11 days, while TAC exposure was ex vivo. Each panel represents a box plot comparing pre-CBD (yellow) and post-CBD (green) conditions stratified according to No CD3/CD28, CD3/CD28 alone (CD3/CD28), and CD3/CD28 with tacrolimus (CD3/CD28 +TAC). The same 3 participants provided samples for all 6 conditions. Because of the volume needed for the assay, biological samples were pooled, and technical replicates were used for statistics. Protein concentrations (pg/mL) were scaled and are shown as *z* score–normalized values. Symbols indicate statistical comparisons: black denotes differences between pre- and post-CBD conditions; blue indicates comparisons between CD3/CD28 –TAC and CD3/CD28 +TAC in the absence of CBD; and red indicates the same comparison in the presence of CBD. Statistical significance is represented as follows: *P < 0.05, **P < 0.01, ***P < 0.001. All *P* values were calculated using the limma package with Benjamini-Hochberg FDR correction. Analytes shown include TNF-α (**A**), lymphotoxin-α (LTA) (**B**), GM-CSF (**C**), IL-4 (**D**), IL-2 (**E**), IL-10 (**F**), IL-1RA (**G**), IL-8/CXCL8 (**H**), MCP-1/CCL2 (**I**), IL-6 (**J**), IL-9 (**K**), and IL-7 (**L**).

To orthogonally support the gene expression changes with functional protein secretion, we assessed ex vivo protein cytokine levels secreted from the lymphocytes, obtained in the same 6 conditions used in the scRNA-Seq analysis. Based on a panel of protein cytokines, we observed a mixed proinflammatory and antiinflammatory response in the post-CBD condition (Figure 7, Supplemental Figure 4, and Supplemental Table 16). For example, we observed reduced TNF-α (Figure 7A) and LTA (Figure 7B) protein expression in the post-CBD condition, consistent with the reduced communication observed in TEM cells in the receptor-ligand analysis (Figure 4, A and B). Likewise, proliferative signals were reduced in the post-CBD participants, including GM-CSF, IL-4, and IL-2 (Figure 7, C–E). Specific antiinflammatory cytokines, such as IL-10 and IL-1RA (antagonists of IL-1), were upregulated after CBD use (Figure 7, F and G). In contrast, inflammatory cytokines (IL-8, CCL2, and IL-9) (Figure 7, H–L) increased after CBD usage in the No CD3/CD28 and CD3/CD28 conditions. IL-6 (Figure 7J) was the most prominent inflammatory cytokine upregulated in the post-CBD participants. Overall, the changes in all effector responses between No CD3/CD28 and CD3/CD28 were less potent (reduced delta) post-CBD, including innate (LTA or IL-12p40), intermediary innate-adaptive Th1 (TNF), adaptive (IL-2), and Th2 (IL-4). In contrast, the delta change of IL-10 was increased post-CBD, consistent with the relative increase in Treg population (for the CD3/CD28 condition; Figure 2D) and *KLF2^+^* T cells (Figure 4D).

We next examined the effects of tacrolimus in addition to the CD3/CD28 condition with and without CBD to serve as a positive control (tacrolimus alone) in order to understand the combined effects of CBD and tacrolimus on cytokine production. Most cytokine expression was reduced following tacrolimus exposure. We observed an expected reduction in proliferative signals such as IL-2, GM-CSF, TNF-α, and LTA in both the CD3/CD28 pre-CBD and post-CBD conditions. We also observed a decrease in IL-10 post-CBD, but CCL2, IL-6, and IL-8 were still upregulated after CBD administration, even with combined tacrolimus exposure (Figure 7).

### Flow cytometry validation of lymphocyte populations.

**Figure 8:**
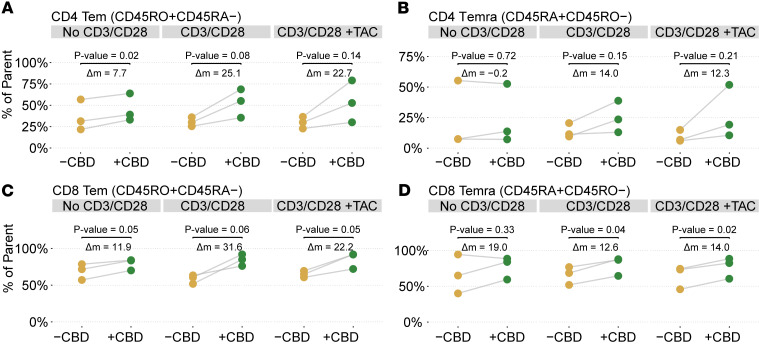
Flow cytometric analyses of T lymphocyte cell subsets with corresponding statistical comparisons. Flow cytometry data for *N* = 3 participants illustrate the changes in cell type proportion pre- and post-CBD. Identified cells include CD4^+^ CD45RO^+^CD45RA^–^ (T effector memory [TEM]) (**A**), CD4^+^ CD45RA^+^CD45RO^–^ (TEMRA) (**B**), CD8^+^ TEM (**C**), and CD8^+^ TEMRA (**D**) T cell populations, each under pre- and post-CBD conditions across 3 experimental groups: No CD3/CD28, CD3/CD28 alone (CD3/CD28), and CD3/CD28 with tacrolimus (CD3/CD28 +TAC). Significance was determined by a paired, 2-tailed *t* test.

To validate the increase in effector memory T cells observed by scRNA-Seq and determine the upregulation of TEM/TEMRA cells by protein markers, we characterized the T cell distribution pre- and post-CBD with flow cytometry (*N* = 3 participants; Figure 8). Cells were first gated as Live/Dead^–^CD45^+^ (viable leukocytes), followed by singlet selection using FSC-H versus FSC-A to exclude doublets. Live single lymphocytes were further classified into CD3^+^CD4^+^ (CD4 T cells) and CD3^+^CD8^+^ (CD8 T cells). Gating of CD8^+^ and CD4^+^ TEM cells was established using CD8 or CD4 and a combination of CD45RO, CD45RA, and other markers (Supplemental Figure 5). The TEM cell distribution by flow cytometry mirrored that of the scRNA-Seq datasets. CD4^+^ TEM cells (CD45RO^+^CD45RA^–^) were upregulated post-CBD in the No CD3/CD28 and CD3/CD28 conditions (Figure 8, A and B). CD8^+^ TEM and TEMRA (CD45RO^–^CD45RA^+^) cells were increased in frequency post-CBD in most conditions (No CD3/CD28, CD3/CD28, and CD3/CD28 +TAC; Figure 8, C and D). We also identified a concomitant reduction in CD4 lymphocytes, consistent with the reduced proliferation in the scRNA-Seq dataset (Supplemental Table 17). With tacrolimus administration, we identified expected changes in cell type distribution (Supplemental Table 17). However, CD8^+^ TEM and TEMRA cells were still relatively more abundant in the tacrolimus post-CBD condition compared with the tacrolimus pre-CBD condition (both *P* < 0.05). Because anti-CD3/anti-CD28 stimulation induces strong T cell receptor engagement, surface CD3 expression is known to be internalized in activated cells (23–27). As a result, gating solely on CD3^+^ events would exclude these highly activated T cells. To confirm that these cells retained T cell identity, we compared unstimulated and stimulated samples from the same participants. Unstimulated cells showed concordant CD3 and lineage marker expression (CD4, CD8, FoxP3), whereas stimulated cells lost surface CD3 but maintained lineage markers, consistent with CD3 internalization rather than loss of T cell identity (Supplemental Figure 6).

## Discussion

Cannabidiol (CBD) has gained widespread popularity and is now readily available without a prescription, raising important questions regarding its potential immunomodulatory effects in vulnerable populations such as allograft recipients. CBD is often regarded as safe owing to its lack of psychoactive and addictive properties (28); however, its FDA drug label includes warnings for hepatotoxicity and an increased risk of infections (29). Prior clinical trials have examined the effect of CBD in human populations with multiple sclerosis (30, 31), revealing improvement in spasticity or other related neurologic symptoms. However, mechanistic insights were not proffered as to whether CBD’s immunomodulatory or neuromodulatory effects drove these improvements. An additional study examined flow cytometry of NK cells in response to 8 weeks of CBD at a very low dose of 50 mg daily (32). Clinical studies addressing the CBD-induced immune changes in humans are still lacking. Many studies in animal or cell culture model systems have shown marked immunosuppressive effects of CBD (11, 33–36). For example, one study examined the immunosuppressive effects on peripheral blood mononuclear cells of individuals with psoriasis vulgaris (37), but a key difference is that CBD was only administered in vitro to cultured cells from these individuals. In contrast, our study is a clinical trial in which participants received oral CBD twice daily to reach a steady state, better reflecting the effects of the in vivo drug.

Our study suggests that CBD, when administered to human participants under steady-state conditions, exerts concentration-dependent immunomodulatory effects. We deeply profiled immune cell populations before exposure (pre-CBD) and at steady state (post-CBD) using single-cell RNA sequencing. A key finding was a significant shift in the proportion of effector memory T cells, particularly the TEMRA phenotype, post-CBD. This shift is likely multifactorial. One factor is enhanced regulatory activity as supported by increased IL-10 cytokine expression, increased Treg-to-TEM-cell ligand-receptor signaling through *LCK* and *HLA* genes/proteins, and a modest increase in the *FOXP3^+^* Treg population in the scRNA-Seq dataset. Prior studies have also demonstrated that CBD induces T regulatory lymphocytes (38). In our study, the upregulation of *KLF2^+^* T cells may similarly contribute to a quiescent T cell and/or TEMRA phenotype (21, 39). A second potential factor is increased TEMRA/TEM terminal differentiation, potentially through the upregulated IL-6. The cytokine IL-6 can promote the formation of effector cells that subsequently become TEM cells (40), and CBD is known to stimulate IL-6 release (41). Our clinical study confirmed the upregulation of IL-6 secretion and flow cytometric increases in TEMRA/TEM cells post-CBD. A third factor in the relative TEMRA/TEM upregulation is reduced proliferation and reduced total cell count of all cells. We observed a relative decrease in B CD70^hi^ and proliferating T cells through the antiproliferative and cytotoxic effects of CBD. In contrast, TEMRA cells may persist longer under CBD conditions. TEMRA cells must first move to RO status, then express the death receptor (e.g., FAS), then move to an IL2RA or subunit rearrangement status to increase their effector function before dying. The upregulated Treg activity may slow this process relative to the reduced proliferation of other T cells. Future mechanistic studies will be required to ascertain the relative contribution of each of these factors and others to the TEMRA/TEM shift post-CBD. In summary, the results suggest that CBD influences T cell differentiation and memory formation, altering the immune balance.

CBD exposure reduced the proliferation of both T and B CD70^hi^ cells, consistent with prior reports of CBD’s antiproliferative effects (10, 11, 42). The effect is potentially secondary to the downregulation of cytokines and receptors such as IL-2 for T lymphocytes and IL-4 and CB2 for B lymphocytes (22, 43). In our study, *CNR2* (CB2) gene expression was reduced post-CBD. CB2 receptors are pre-proliferative and upregulated during inflammation, so *CNR2* downregulation is consistent with an antiinflammatory effect post-CBD (44). A pre-specified goal of this study was to determine whether CBD and tacrolimus held additive or synergistic immunologic effects. IL-2 protein and *IL2* gene expression were suppressed by CBD, aligning with the known effects of tacrolimus, which also reduced IL-2 production. This suggests that CBD may share mechanistic overlap with tacrolimus, raising essential considerations for its use in allograft recipients or other immunocompromised populations. The relative decrease in B CD70^hi^ and proliferating T cells, with increased proportions of TEMRA/TEM cells, suggests mixed inflammatory and antiinflammatory effects. Thus, our study demonstrates that CBD alters immune cell distribution and signaling in a nuanced and concentration-dependent manner.

In our assays, we selected against myeloid cells to better study CBD’s effect on lymphocyte populations. While we note a relative increase in myeloid cells in participants post-CBD, only a small number of myeloid cells were retained in the scRNA-Seq populations (8,370 cells across all conditions compared with 450,774 lymphocytes). Thus, we refrain from conclusions regarding the effects of CBD on myeloid cells given our experimental design that included positive lymphocyte selection. Prior investigations have focused on myeloid cells, identifying expression and phenotype changes. For example, certain studies have found that CBD induces an antiinflammatory macrophage phenotype (36, 37). Other studies have shown that CBD induces apoptosis in macrophages (45, 46). We also identified a second clinical study that administered CBD at a daily dose of about 1 mg/kg/d and reported an antiinflammatory effect of CBD in the myeloid cell clusters (2,277 total myeloid cells) (47).

Our study had certain limitations. First, this is a secondary analysis of individuals who participated in a pharmacokinetic clinical study (5). Thus, we were limited in the blood and sampling that could be performed outside the pharmacokinetic time points. Further, all single-cell power calculations were performed post hoc, but the large quantity of cells (459,144) ensured adequate power to detect shifts in lymphocyte distribution. TEM and TEMRA cells are challenging to distinguish within scRNA-Seq data; thus, we performed cytokine and flow cytometry analyses in a subset of participants to support the findings on an orthogonal protein level. Because of the prospective nature of this study, we cannot reinterrogate all individuals who previously completed the trial using every methodology. Next, the dose of CBD administered (10 mg/kg/d total dose) is an FDA-approved dose, but higher than those of typical over-the-counter products (range: 0.1–5.6 mg/kg/d) (48). An additional limitation is that we assessed immune cell expression from cells obtained from the blood in healthy volunteers, not intrarenal immune cells of allograft recipients.

In conclusion, CBD appears to hold mixed pro- and antiinflammatory effects. We believe the data presented here will be informative to transplant physicians who encounter patients who take CBD. It is now well known that CBD and tacrolimus interact pharmacokinetically, which can lead to unsafe elevations in tacrolimus concentration (4). However, even when tacrolimus levels are fixed at 5 ng/mL, we have demonstrated important changes in cytokine signaling and immune cell distribution. These may hold downstream ramifications for prevention of allograft rejection and resistance to opportunistic infections. Inflammatory IL-6 cytokine secretion and TEMRA/TEM cell proportion were upregulated in the post-CBD condition, regardless of the presence of tacrolimus. In particular, the changes in TEM proportion were concentration dependent, suggesting that providers must be aware of the total daily dose of CBD their patients take. Future studies must examine long-term outcomes in transplant recipients.

## Methods

*Sex as a biological variable*. Male and female participants were enrolled in the phase I clinical trial (Supplemental Table 1). For this study, sex was not considered as a variable owing to sample size.

*Study design and participants*. The study is a secondary analysis of mechanistic molecular data acquired from healthy human volunteers who completed a phase I clinical study (NCT05490511). Details of the trial design, eligibility criteria, interventions, and clinical procedures have been described (5). Participants (*N* = 23) represent a predefined subset of the study population. Individuals were aged 18–65 without medical comorbidities or predicted drug-drug interactions (Supplemental Table 1). Blood samples were acquired at 2 time points. The first sample was acquired before the first CBD dose (pre-CBD). The second sample was acquired before the morning CBD dose on day 12 after 11 days of CBD titration (post-CBD). Most participants received Epidiolex (CBD oral solution, GW Pharmaceuticals) at a dose of 2.5 mg/kg twice daily for 3 days, followed by 5 mg/kg twice daily for 11 additional days (14 total days), which is within the range of titration approved by the FDA. Blood samples for pharmacokinetic analysis were collected on day 12 to 14, after 11 full days of CBD administration, at the following time points: 0 hours, 0.33 hours, 0.66 hours, 1 hour, 2 hours, 4 hours, 6 hours, 12 hours, 24 hours, and 48 hours.

*Blood sample processing*. The peripheral blood mononuclear cells (PBMCs) were acquired on day 1 (pre-CBD) and day 12 (post-CBD) from participants and then underwent 72 hours of ex vivo preparation for the following 6 conditions involving CBD, tacrolimus (TAC), and CD3/CD28 stimulation (CD3/CD28): (a) No CD3/CD28 / –CBD / –TAC, (b) CD3/CD28 / –CBD / –TAC, (c) CD3/CD28 / –CBD / +TAC, (d) No CD3/CD28 / +CBD / –TAC, (e) CD3/CD28 / +CBD / –TAC, and (f) CD3/CD28 / +CBD / +TAC. Blood samples were diluted 3-fold using Dulbecco’s phosphate-buffered saline (DPBS) (Corning Inc., 21-031-CM) with 1% fetal bovine serum (FBS) (Gibco, A3840101) and added on top of 15 mL of Ficoll-Paque PLUS (Cytiva, 17144002) in a tube, which was centrifuged at 600*g* for 30 minutes at room temperature. The buffy coat containing PBMCs atop the Ficoll layer was pipetted into a tube and washed with DPBS with 1% FBS twice with repeated centrifuging and discarding of supernatant. Finally, the cell pellets were resuspended in 10 mL of RPMI 1640 medium (Roswell Park Memorial Institute, Corning Inc., 10-040-CM) with 1% FBS and 1% penicillin-streptomycin to which 50 μL of phytohemagglutinin-L (MilliporeSigma, 11249738001) was added for preferential lymphocyte selection. Each participant’s cells were transferred to a T25 flask and incubated at 37°C and 5% CO_2_ for 24 hours of ex vivo primary culture.

After 24 hours, floating cells not affixed to the T25 flask were harvested and centrifuged at 600*g* for 5 minutes at room temperature. Subsequently, the supernatant was discarded, and the cells were resuspended in RPMI. Samples of each participant were divided into 3 conditions and incubated in T25 flasks or 96-well plates at 37°C and 5% CO_2_ for 48 hours. The first condition (CD3/CD28) received CD3/CD28 lymphocyte stimulation at 25 μL/mL (ImmunoCult Human CD3/CD28 T Cell Activator, STEMCELL Technologies, 10971). The second condition (CD3/CD28 +TAC) received CD3/CD28 lymphocyte stimulation with tacrolimus at a concentration of 5 ng/mL (MedChemExpress, HY-13756/CS-1507). A third condition (No CD3/CD28 / baseline) did not have CD3/CD28 activators or tacrolimus added to the samples.

After 72 hours, cells were retrieved. Cells in T25 flasks were used for scRNA-Seq, flow cytometry, or cytokine measurement. The cells were centrifuged at 600*g* for 5 minutes at room temperature, washed, and resuspended in RPMI for downstream processing. Cells from 96-well plates were used for the cell proliferation assay.

*Luminescence assay*. Cell viability was measured in cells using CellTiter-Glo (Promega Corp., catalog G7570) on 96-well plates to quantify cell proliferation by adenosine triphosphate (ATP) detection using a Spectramax M5 system coupled with SoftMax Pro 5.2 (Molecular Devices). Pre-CBD (*N* = 17) and post-CBD (*N* = 18) values were calculated as a ratio of ATP detection in the stimulated (CD3/CD28) condition normalized by the non-stimulated (No CD3/CD28) condition. Ratios were separately calculated with and without tacrolimus. Conditions were compared using a 2-tailed *t* test (Supplemental Table 2).

*PBMC single-cell RNA sequencing*. For each of the 6 conditions in Supplemental Table 1, library preparation was performed using the 10x Genomics Chromium Single Cell v3.1 NextGem platform. cDNA synthesis and amplification were performed according to the 10x Genomics protocols to ensure optimal yield and specificity. Quality control measures were applied at multiple stages to assess library integrity, concentration, and fragment size; sequencing was conducted on the Illumina NovaSeq 6000 platform. Paired-end reads were generated with a length of 100 bp (PE100), and sequencing depth was optimized according to experimental requirements. Fifty-nine single-cell RNA sequencing (scRNA-Seq) experiments were sequenced across multiple runs to achieve sufficient coverage for downstream analyses (Supplemental Table 1). Data were obtained from 12 participants who provided pre-CBD scRNA-Seq samples, 8 of whom also offered post-CBD scRNA-Seq samples, which passed quality control. Two participants withdrew before the post-CBD sample acquisition, and 2 samples did not pass quality control. Alignment and quantification were accomplished with Cell Ranger (v7.1.0, 10x Genomics) using default options and reference genome GRCh38-2020-A. The BPCells (v0.2.0) package (49) converted the matrices in the Hierarchical Data Format h5 to bitpacked compressed format stored as binary files on a hard drive. We used Seurat (v5.1.0) to create a Seurat object for each sequencing sample. Cells with mitochondria reading over 25% or fewer than 500 genes were removed. All scRNA-Seq samples were merged, and the cells were annotated by Azimuth (v0.5.0) using the PBMC_ref_4546839 in Zenodo (https://zenodo.org/records/4546839) (50). For the 6 conditions, the scRNA-Seq object was split into layers by sample, with the following methods: NormalizeData, FindVariableFeatures, ScaleData, and IntegrateLayers, finally using the method HarmonyIntegration with the harmony (v1.2.0) tool performed to integrate the samples.

Two additional participants provided snRNA-Seq at the post-CBD condition, which only increased the sample size for comparisons between pharmacokinetics and cell type proportion (Figure 3). These samples were analyzed separately and not included in the Seurat object or other scRNA-Seq analyses beyond those of Figure 3. Cell annotations were performed in an analogous way to that of scRNA-Seq. A principal component analysis for cell type proportion did not reveal differential clustering of the scRNA-Seq and snRNA-Seq samples.

*Cell proportion quantification*. The proportions of each cell type across conditions were calculated to assess differences in cell distribution. Cells in each condition were grouped into cell type, and the count of each cell type was divided by the total cell count within that condition to determine the cell type–specific proportions. Odds ratios were calculated by comparison of the proportions between conditions (e.g., A and B) for each cell type, specifically by computation of

Significance of the observed difference was evaluated using a Fisher’s exact test, with a *P* value less than 0.05 indicating significance.

### Confidence intervals (95% CI) for the odds ratios were calculated by taking the natural logarithm of the odds ratio (OR) and applying the following formula:

where *a*, *b*, *c*, and *d* represent the cell counts in each condition matrix for the analyzed cell type. The 95% CI was then exponentiated to obtain the final interval on the odds ratio scale, and results were visualized through a forest plot.

#### Pharmacokinetics of CBD at steady state.

CBD steady-state concentrations were obtained using a liquid chromatography–tandem mass spectrometry assay (5). Ten participants provided 10 pharmacokinetic (PK) time points with CBD concentrations. The data were analyzed by noncompartmental analysis using Phoenix WinNonlin version 8.4 (Certara LP). The following pharmacokinetic parameters were derived: concentration at time 0 hours (C_0h_, ng/mL), maximum blood concentration (C_max_, ng/mL), time to C_max_ (T_max_, hours), area under the plasma concentration–time curve within a dosing interval (AUC_0–τ_, h·ng/mL), concentration at the end of the dosing interval (C_τ_, ng/mL), minimum concentration (C_min_, ng/mL), and time at C_min_ (T_min_, hours).

Descriptive statistics were computed for each parameter, including mean, median, minimum (min), maximum (max), standard deviation (SD), and coefficient of variation (CV%). Given the small sample size, a *t* distribution was used to estimate the 95% CI. The confidence coefficient was determined using the *t* critical value for a 95% confidence level with 9 degrees of freedom. The margin of error was calculated by multiplication of the *t* critical value by the standard error. The 95% CI was computed as mean ± SEM, providing upper and lower limits.

#### PK correlation to cell proportion.

To assess the correlation between sc/snRNA-Seq and PK parameters, we analyzed data from 10 participants (*N* = 10) who had measurements available for both data types under the post-CBD (CD3/CD28 / +CBD / –TAC) condition. We applied linear regression using the lm() function in R to model the relationship between an independent variable (C_0h_) and the dependent variable (TEM proportion). The C_0h_ parameter was selected for comparison because it is the same concentration time point at which participants had their sc/snRNA-Seq sample drawn. Pearson’s correlation was used to evaluate the strength of the association, with statistical significance at *P* < 0.05. The *R*^2^ value from the regression indicates the proportion of variance in cell type proportion explained by C_0h_.

#### Pathway analyses.

Differentially expressed genes (DEGs) were identified across conditions of interest using the default settings of RunPresto from the SeuratWrappers (v0.3.5) and presto (1.0.0) packages (51). DEGs were filtered to include only genes with a Wilcoxon’s adjusted *P* value less than 0.05 after Benjamini-Hochberg correction and were ranked by the absolute value of log fold change (*|*avgLogFC*|*). To identify the gene set enhanced and suppressed by CBD, DEGs from No CD3/CD28 –CBD versus CD3/CD28 –CBD were plotted on the *x* axis, and DEGs from CD3/CD28 CBD versus CD3/CD28 –CBD were plotted on the *y* axis. The genes in quadrant 2 (Q2) were enhanced by CBD, and the genes in quadrant 4 (Q4) were suppressed by CBD. The top 300 ranked by Euclidean distance from the origin were annotated and used for pathway enrichment analysis. Pathway enrichment analysis was conducted using pathfindR (v2.4.1) (52). The enrichment parameters for run_pathfindR were configured with the KEGG gene sets and the BioGRID protein interaction network, allowing for pathways with a minimum of 1 and a maximum of 500 genes, and the adjusted *P* value less than 0.05. The enriched pathways were screened for the presence of genes in pathways related to CBD receptors (3) and receptor-ligand pairs identified in the cell-cell communication analyses (Supplemental Tables 7, 11, and 13).

#### Pathway analyses and concentration association.

To assess associations between pathway activity and treatment, we calculated per-participant mean pathway scores within the target cluster and compared them with the mean of all other cells (cell type target/all other cell types). These ratios were correlated with baseline concentration (C_0h_) using Pearson’s correlation. *P* values were adjusted for multiple testing using the Benjamini-Hochberg method. Figure 3, D–G, shows a volcano plot demonstrating the association between pathway activity ratios (target cluster vs. rest) and baseline concentration (C_0h_).

#### Immune cell-cell communication.

Analysis of scRNA-Seq datasets was performed by CellChat (v2.1.2) (53) to assess the cell-cell communication across cell types and compare the pre-CBD and post-CBD stimulated conditions (Supplemental Table 8).

#### PBMC cytokine multiplex assay.

Cells from the 6 conditions were used in a multiplex assay. The supernatant of lymphocytes was aliquoted in 1.5 mL tubes and kept at –80°C until use. Samples from 3 participants then underwent multiplex assays using ProcartaPlex Human Cytokine/Chemokine/Growth Factor Convenience Panel 1 (Invitrogen, Thermo Fisher Scientific Inc., EPXR450-12171-901). The samples were pooled by condition, and comparisons were made between the pre-CBD and post-CBD time points (Supplemental Table 1).

For the multiplex assay, samples were thawed on ice and assayed by Luminex xMAP technology (Invitrogen, Thermo Fisher Scientific, 501125916) for 45 human cytokines and chemokines (Supplemental Table 17). Cytokine concentrations were quantified from culture supernatants with the 45-plex Luminex assay and exported as pg/mL values. The genes (cytokine) × samples (well) expression matrix was analyzed with limma (v3.60.6). A no-intercept design matrix encoded every experimental condition: (a) No CD3/CD28 / –CBD / –TAC, (b) CD3/CD28 / –CBD / –TAC, (c) CD3/CD28 / –CBD / +TAC, (d) No CD3/CD28 / +CBD / –TAC, (e) CD3/CD28 / +CBD / –TAC, (f) CD3/CD28 / +CBD / +TAC.

Differential cytokine abundance was tested with limma’s moderated *t* statistic, controlling for the false discovery rate (FDR) with a Benjamini-Hochberg adjustment at FDR < 0.05 (54).

#### PBMC flow cytometry.

After 72 hours of primary culture, Petri dishes containing both attached and floating cells were scraped, and the culture media containing the cells were centrifuged at 1,600*g* for 5 minutes. The cell pellets were transferred to 2 mL tubes, and CryoStor CS10 was added. The cells were placed on ice for 10 minutes, stored overnight in a deep freezer within a slow-freeze container, and finally moved to liquid nitrogen until used for flow cytometry analyses. Thawed cells were resuspended in fresh culture medium (RPMI supplemented with 1% FBS and 1% penicillin-streptomycin) and centrifuged (350*g*, 7 minutes, 4°C), and supernatants were aspirated. Cells were incubated with Live/Dead Olive dye (Invitrogen, Thermo Fisher Scientific, L34977) (1:1,000 dilution in DPBS) for 30 minutes at 4°C (light-protected) and washed by centrifugation (350*g*, 7 minutes, 4°C), and supernatants were removed. Fc receptors were blocked with FcBlock (Invitrogen, eBioscience, Thermo Fisher Scientific, A53011) (5 μL/tube, 10 minutes, 4°C). Antibody cocktails, single stains, and fluorescence-minus-one controls were prepared and added to cell suspensions. After 30 minutes of incubation (4°C, light-protected), cells were washed with 2 mL flow cytometry staining buffer (PBS plus 2% FBS) and centrifuged (350*g*, 7 minutes, 4°C).

For intracellular staining, cells were fixed and permeabilized using IC Fixation/Permeabilization Buffer (Invitrogen, eBioscience, Thermo Fisher Scientific, 00-5523-00) (20 minutes, room temperature, light-protected), washed with 2 mL permeabilization buffer, and centrifuged (350*g*, 7 minutes, 4°C). Permeabilized cells were resuspended in 100 μL permeabilization buffer, incubated with intracellular antibody cocktails (20 minutes, room temperature, light-protected), rewashed with 2 mL permeabilization buffer, and finally resuspended in flow cytometry staining buffer for acquisition. Single antibody staining controls were prepared using UltraComp eBeads Plus (Invitrogen, Thermo Fisher Scientific, 01-3333-41). All samples were analyzed using the Cytek Aurora System. Flow cytometry population frequencies were normalized by rarefying of subpopulation counts to the minimum CD3^+^ T cell count per condition. Differential abundance between conditions was assessed using 2-sided Fisher’s exact tests on 2×2 contingency tables, followed by Benjamini-Hochberg correction for multiple comparisons (55).

For gating, live singlet lymphocytes were selected by forward scatter area and forward scatter height with exclusion based on the viability dye. Total T cells were identified as CD3^+^ events and split into CD4^+^ and CD8^+^ lineages. Within the CD8^+^ compartment we resolved memory/effector phenotypes on the basis of CD45 isoforms: CD45RO^+^CD45RA^–^ effector memory (TEM), CD45RA^+^CD45RO^–^ (TEMRA), and an intermediate CD45RA^+^CD45RO^–^ pool that was further subdivided into intermediary TEM, TCM (central memory), and naive/TEMRA subsets according to granzyme A, TGF-β, CD127, and CD25 expression. CD4^+^ T cells were partitioned into regulatory (CD25^hi^FoxP3^+^) and non-T-regulatory (CD25^lo^FoxP3^–^) fractions; each fraction was then stratified in an identical CD45RO/CD45RA hierarchy yielding TEM, TCM, intermediary TEM, naive, and TEMRA populations.

#### Statistics.

All analyses were performed using the statistical tests specified in the corresponding figure legends and subsections of Methods. In general, statistical significance was defined as an adjusted *P* value less than 0.05, as appropriate for each analysis. When multiple comparisons were performed, *P* values were corrected for FDR using the Benjamini-Hochberg method.

#### Study approval.

The study was conducted under Institutional Review Board (IRB) approval (Indiana University IRB 12763).

#### Data and code availability.

Data are available within the NCBI’s Gene Expression Omnibus database (GEO GSE303581). All data values underlying the figures and supplemental materials are reported in the Supporting Data Values file. Most code methods were derived from standard packages referenced in Methods.

## Funding support

This work is the result of NIH funding, in whole or in part, and is subject to the NIH Public Access Policy. Through acceptance of this federal funding, the NIH has been given a right to make the work publicly available in PubMed Central.

- National Institutes of Health (NIH)/National Center for Complementary and Integrative Health (R01AT011463).
- NIH/National Institute of General Medical Sciences (NIGMS) (R35GM145383).
- Intramural Research Program of the NIH.
- NIH/NIGMS (T32GM008425 to GCS and JE).

## Supplementary Material

Supplemental data

ICMJE disclosure forms

Supplemental tables 1-17

Supporting data values

## Figures and Tables

**Table 1:**
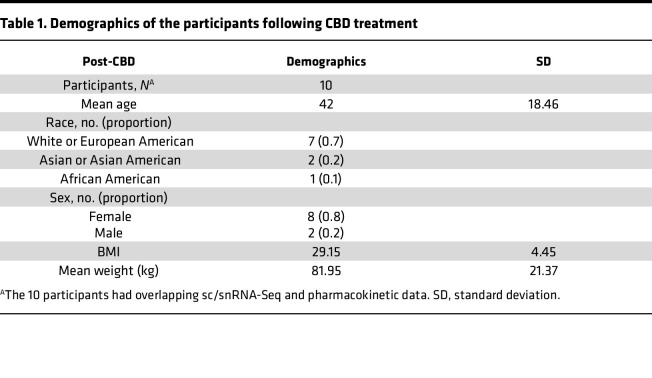
Demographics of the participants following CBD treatment

**Table 2:**
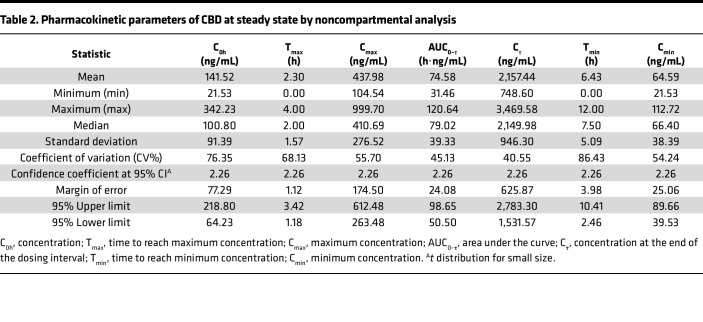
Pharmacokinetic parameters of CBD at steady state by noncompartmental analysis

## Version 1 11/18/2025

In-Press Preview

## Version 2 01/09/2026

Electronic publication
