# Supplementary material for: Cannabidiol exerts antiinflammatory effects but maintains T effector memory cell differentiation in humans
Source: JCI Insight. 2025 Nov 18;11(1):e198590. doi: 10.1172/jci.insight.198590 (PMC12890486; doi:10.1172/jci.insight.198590)
Supplement: Supplemental data [file jciinsight-11-198590-s187.pdf]

Supplemental Figures and Legends

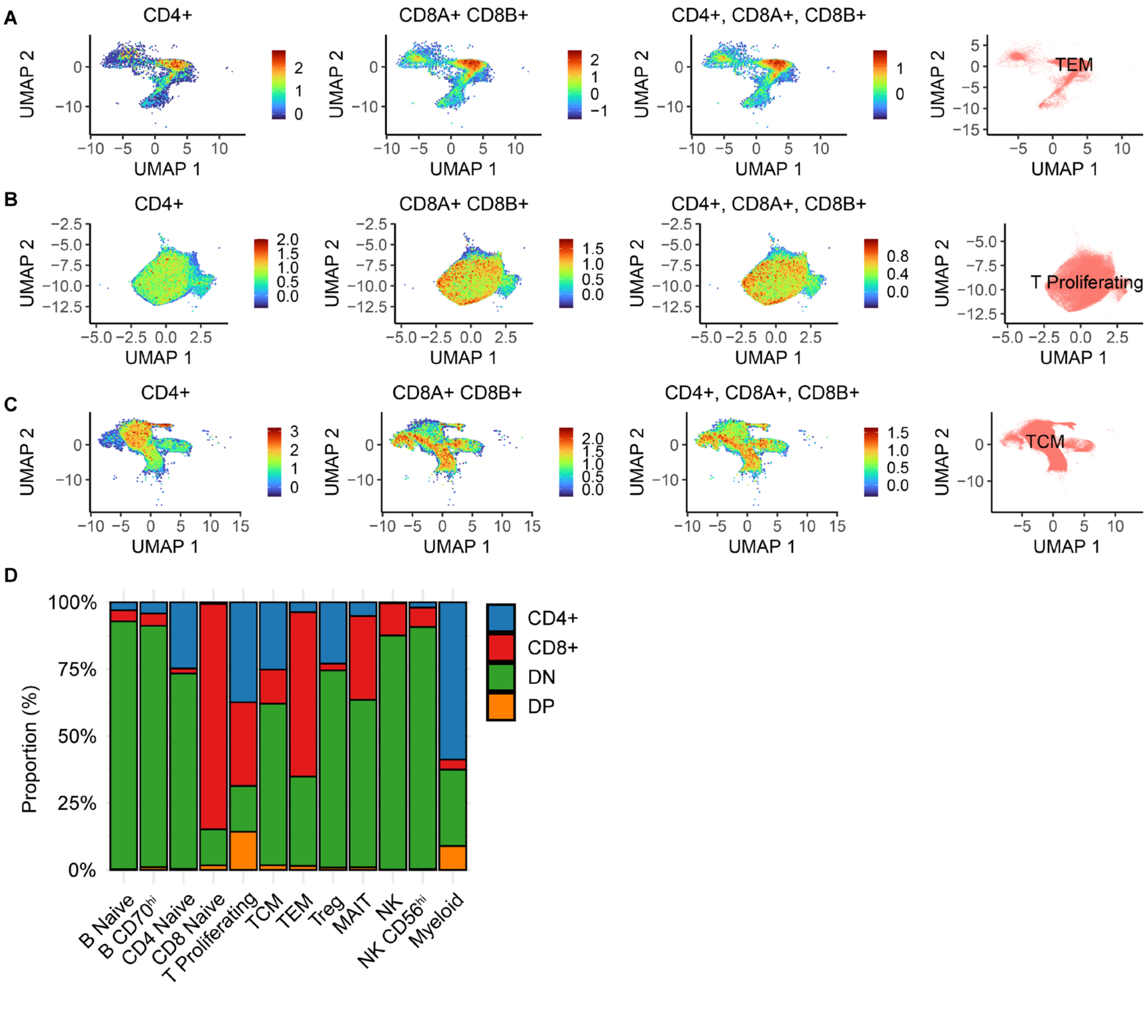

Supplemental Figure 1. Phenotype-Driven Annotation of T-Cell Clusters.

Clusterization of CD8+ and CD4+ Overlap Clustering of T cell populations based on transcriptional profiles. UMAP visualization shows clustering of T cells, with annotations highlighting major phenotypic subsets. In some cases, CD8+ and CD4+ T cells are resolved into distinct clusters, while in others, they are grouped. This indicates that clustering is primarily driven by phenotypic features rather than strict lineage (CD8/CD4) identity, reflecting shared functional or transcriptional programs across subsets. The feature plots are showing this effect occurring in (**A**) T effector memory (TEM), (**B**) T Proliferating and (**C**) T central memory (TCM). (**D**) Subtype composition of T cell clusters. Expression of CD4, CD8A, and CD8B was used to classify cells into four categories: $CD4^+$, $CD8^+$, double-negative (DN), lacking both CD4 and CD8 expression, or double-positive (DP), co-expressing CD4 and CD8. Stacked bar plots display the proportion of these subtypes within each cluster.

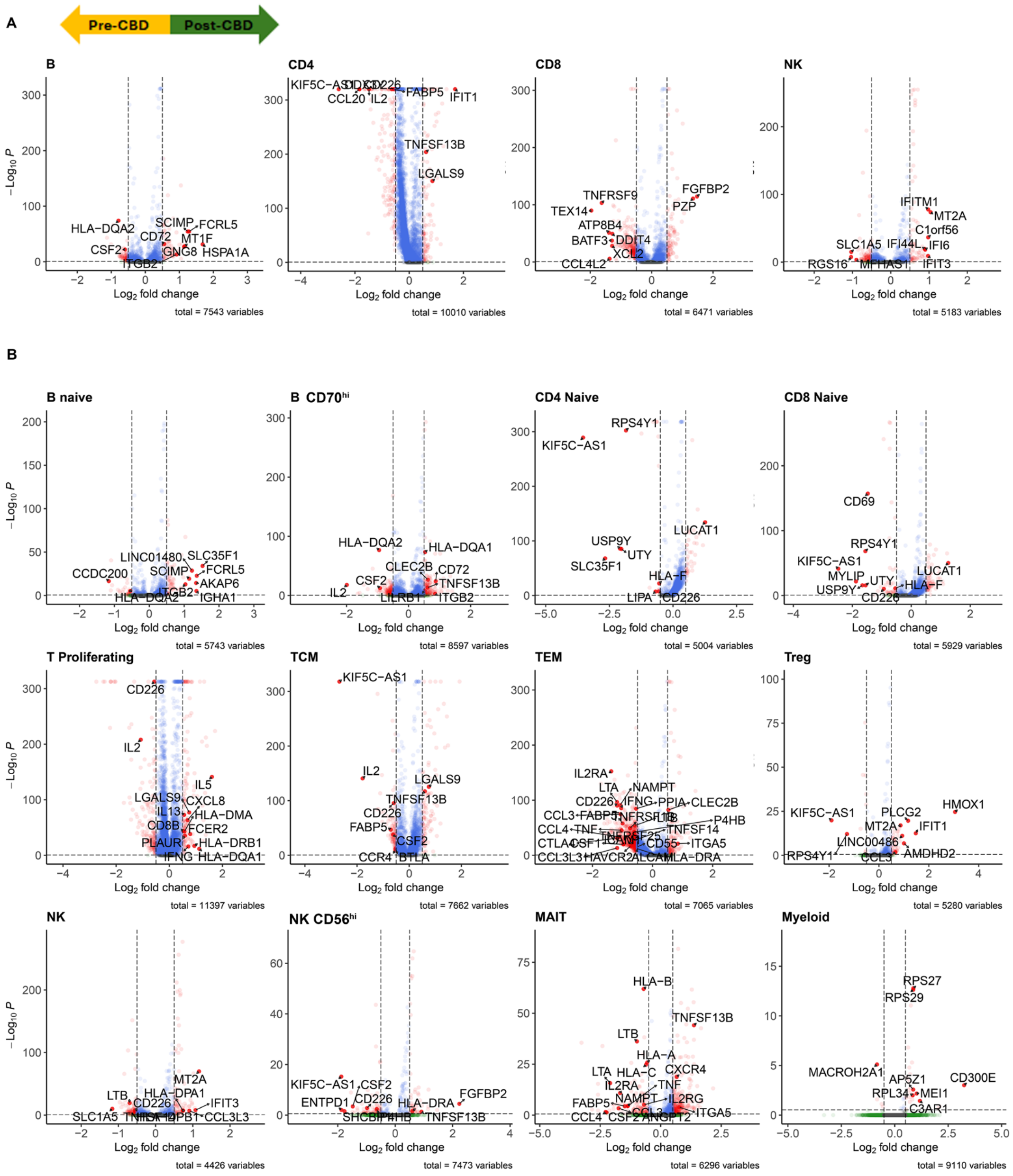

Supplemental Figure 2. Differential expression genes (DEGs) in CD3/CD28 -CBD versus CD3/CD28 +CBD.

The volcano plots show red dots for genes that have average log2FC > 0.5 and an adjusted P-value < 0.05 (Wilcox and Benjamini-Hochberg). Blue dots correspond to adjusted average log2FC < 0.5 and adjusted P-value < 0.05. Green genes correspond to an average log2FC > 0.5 and adjusted P-value > 0.05. Grey dots correspond to an average log2FC < 0.5 and adjusted P-value > 0.05. Negative x-axis genes are downregulated, CD3/CD28 post-CBD, and positive x-axis genes are upregulated CD3/CD28 post-CBD. (**A**) DEGs at annotation Level 1: B, CD4, CD8, NK and Myeloid (**B**) DEGs at annotation Level 2: B naive, B CD70hi, CD4 Naive, CD8 Naive, T Proliferating, T Central Memory (TCM), T effector memory (TEM), T regulatory (Treg), Mucosal-associated invariant T (MAIT), Natural Killer (NK), and Natural Killer CD56+ (NK CD56hi).

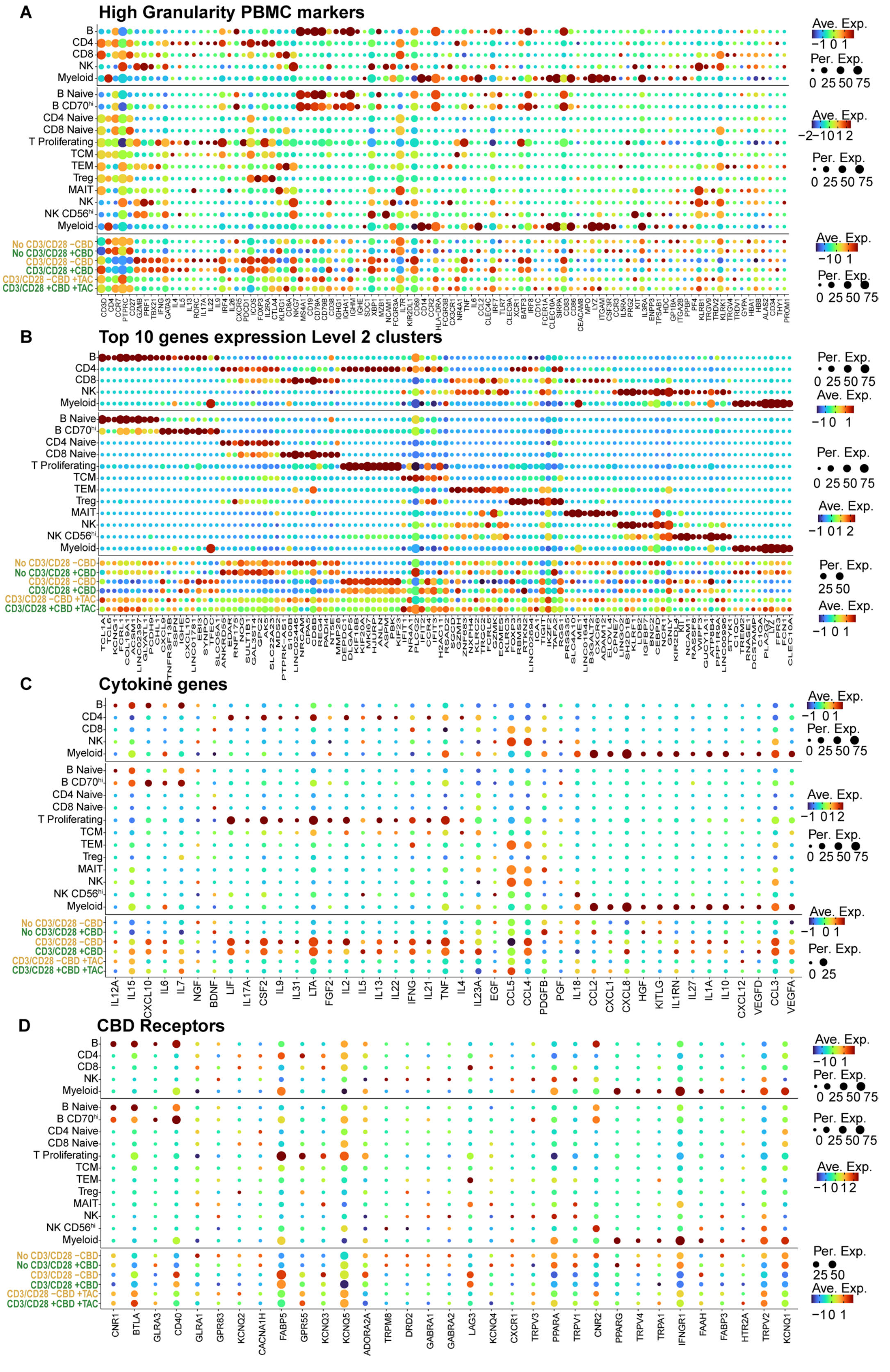

Supplemental Figure 3. Comparative Gene-Set Dotplots Across Three Cell Annotations. The Dot plot shows the expression of the genes across 3 annotation levels: Level 1 (B, CD4, CD8, NK and Myeloid), Level 2 with 12 cell types (B Naïve, B CD70^{hi}, CD4 Naïve, CD8 Naïve, T proliferating, TCM, TEM, Treg, MAIT, NK, NK CD56^{hi} and Myeloid), and Level 3 for the 6 conditions: No CD3/CD28 -CBD, No CD3/CD28 +CBD, CD3/CD28 -CBD, CD3/CD28 +CBD, CD3/CD28 –CBD +TAC and CD3/CD28 +CBD +TAC. (**A**) PBMC markers with high granularity to identify the main immune cell population. (**B**) Top 10 differentially expressed genes (DEGs, unbiased) for each cluster based on Level 2 annotation, but plotted for all three annotation levels. (**C**) cytokine proteins using the respective gene symbol. (**D**) CBD receptors were used in our analyses to filter the pathway analyses.

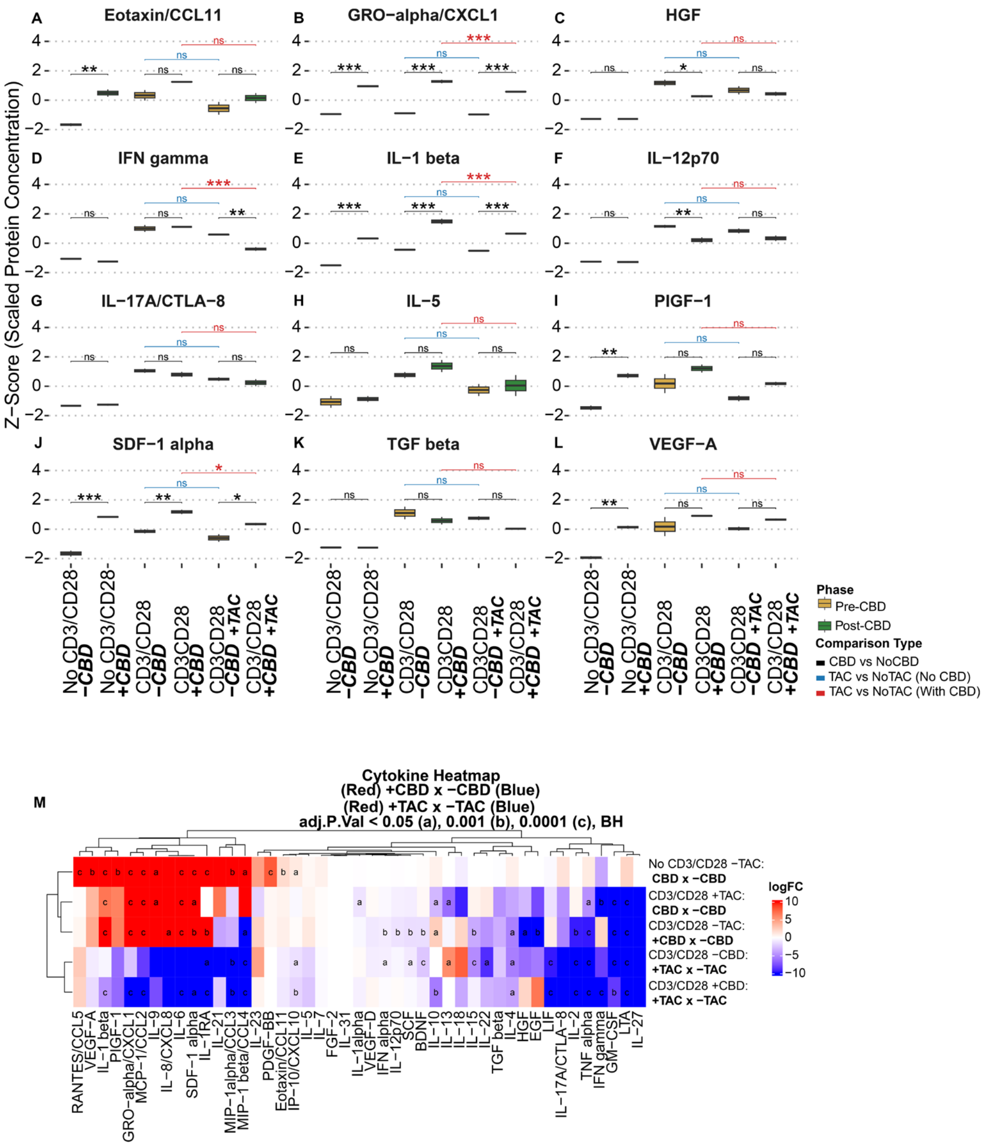

Supplemental Figure 4. Additional cytokine and chemokine levels in the (±CD3/CD28), (±CBD), and (±TAC) conditions.

Each panel presents box plots comparing cytokine levels in the pre-CBD (yellow) and post-CBD (green) conditions across three experimental groups: No Stimulation (No CD3/CD28), Stimulation alone (CD3/CD28), and Stimulation with Tacrolimus (CD3/CD28 + TAC). Protein concentrations are shown as z-score normalized values (pg/ml). Symbols indicate statistical comparisons: black denotes differences between pre- and post-CBD conditions; blue indicates comparisons between CD3/CD28–TAC and CD3/CD28 +TAC in the absence of CBD; and red indicates the same comparison in the presence of CBD. The statistical significance is represented as follows: P-value < 0.05 a, P-value < 0.01 b, P-value < 0.001 c. All p-values were calculated using the limma package with Benjamini–Hochberg false discovery rate (FDR) correction. Analytes shown include: (**A**) EOTAXIN/CCL11, (**B**) GRO-alpha/CXCL1, (**C**) HGF, (**D**) IFN gamma, (**E**) IL-1 beta, (**F**) IL-12p70, (**G**) IL-17A/CTLA-8, (**H**) IL-5, (**I**) PGF1, (**J**) SDF-1, (**K**) TGF beta, and (**L**) VEGF-A. The heat map (**M**) provides an integrated overview of cytokine and chemokine responses across the same experimental comparisons depicted in the box plots. Each row corresponds to a specific group-wise comparison—namely, pre-CBD versus post-CBD, CD3/CD28 –TAC versus CD3/CD28 +TAC in the absence of CBD, and CD3/CD28 –TAC versus CD3/CD28 +TAC in the presence of CBD—aggregated across all measured cytokines. This visualization enables the identification of coordinated immunological patterns and potential treatment-related effects, offering a comprehensive, systems-level perspective on the modulatory impact of CBD and Tacrolimus.

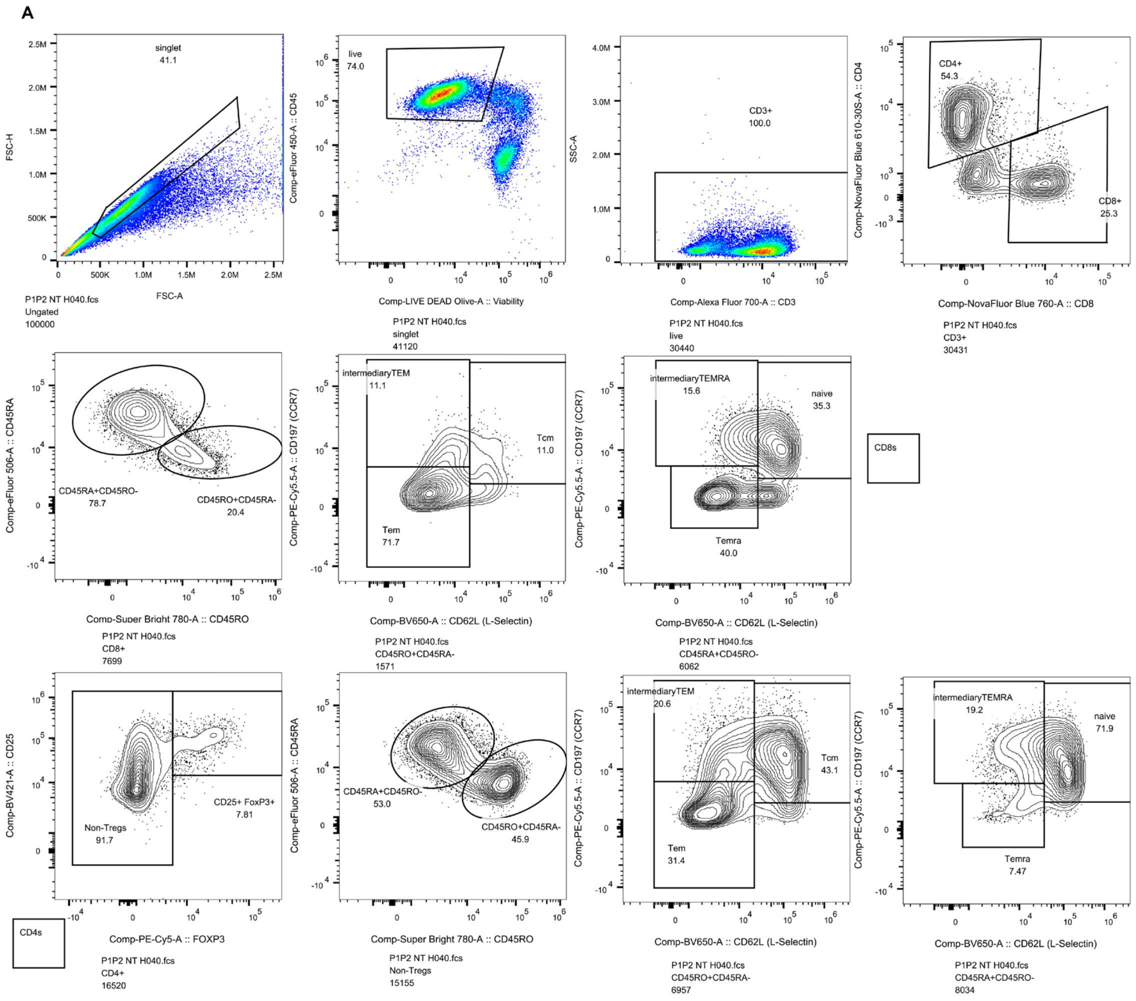

Supplemental Figure 5. Representative flow-cytometry gating strategy used to delineate T-cell subsets.

(**A**) Live singlet lymphocytes were first selected by FSC-A × FSC-H and exclusion of the viability dye. Total T cells were identified as CD3+ events and subsequently split into $CD4^+$ and $CD8^+$ lineages. Within the CD8 compartment, we resolved memory/effector phenotypes on the basis of CD45 isoforms: CD45RO+ CD45RA- effector-memory (Tem), $CD45RA^+$ $CD45RO^-$ (Temra), and an intermediate $CD45RA^+$ $CD45RO^-$ pool that was further subdivided into intermediary Tem, Tcm (central memory) and naive/Temra subsets according to granzyme-A, TGF-β, CD127 and CD25 expression. CD4 T cells were first partitioned into regulatory (CD25^high $FoxP3^+$) and non-T-regulatory (CD25^low $FoxP3^-$) fractions; each fraction was then stratified in an identical CD45RO/CD45RA hierarchy yielding Tem, Tcm, intermediary Tem, naive and Temra populations. Median fluorescence of CD127 (IL-7Rα) and CD25, as well as granzyme-A^+ and TGF-$β^+$ functional overlays, are displayed for the indicated gates. Percentages shown correspond to the frequency of each subset within its parent population. Abbreviations: Tem, effector memory; Temra, terminally differentiated effector memory re-expressing CD45RA; Tcm, central memory; cTreg, conventional regulatory T cell. The participant in the condition No CD3/CD28 -CBD -TAC (P1P2 NT H040) starts with 30440 CD3+ cells where 54.3% is CD4+ and 25.3% is CD8+.

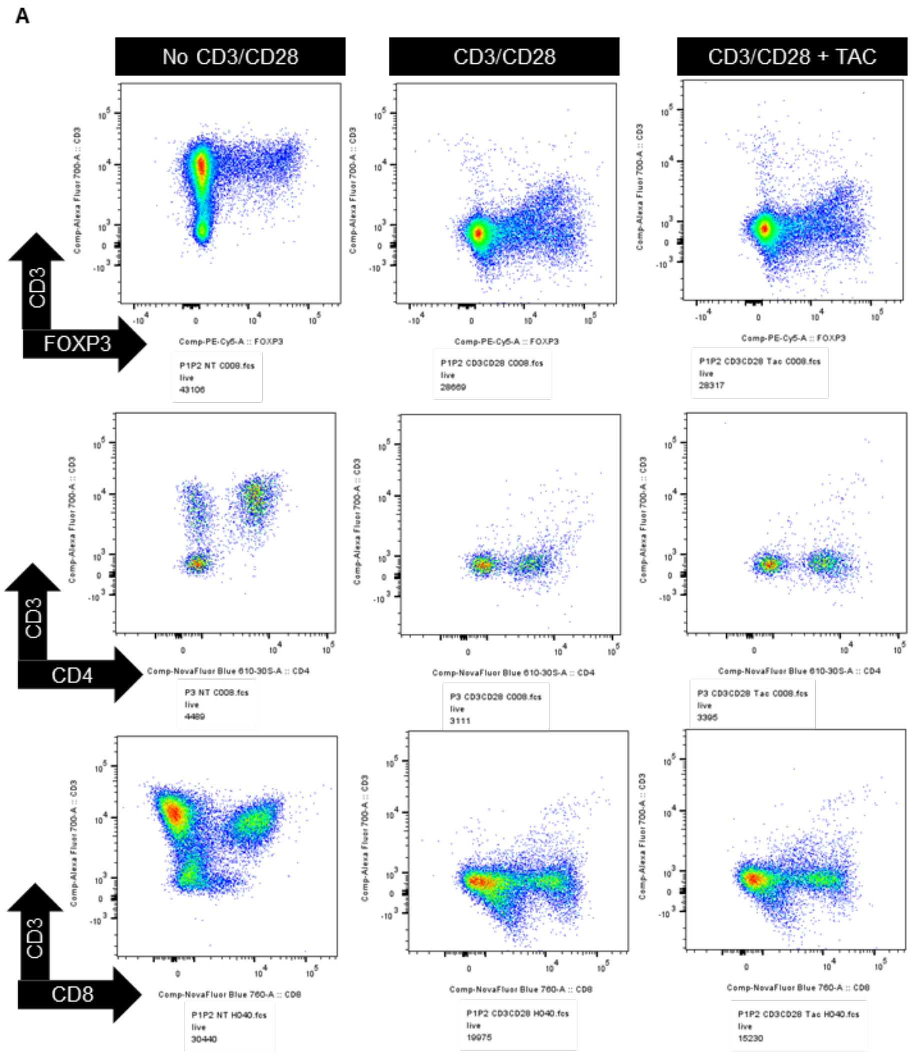

Supplemental Figure 6. CD3 internalization following T-cell activation.

Representative flow cytometry plots showing surface CD3 expression with FOXP3, CD4, and CD8 in paired samples under three conditions: No CD3/CD28 (left), stimulated with CD3/CD28 (middle), and stimulated with CD3/CD28 + tacrolimus (right). In unstimulated samples, FOXP3+, CD4+, and CD8+ cells coexpress CD3, confirming T-cell identity. After stimulation, CD3 surface intensity decreases across all subsets, consistent with receptor internalization following T-cell activation. This effect persists with tacrolimus treatment, although activation intensity differs.

Supplemental Tables

Supplemental Table 1. Participants Stratified Across Experimental Conditions and Technologies.
Supplemental Table 2. CellTiter-Glo® Data.
Supplemental Table 3. Differentially Expressed Genes (DEGs) Across the Three Annotation Levels.
Supplemental Table 4. Cell Proportion Forest Plot Pre-CBD versus Post-CBD.
Supplemental Table 5. Volcano Plots CD3/CD28 +CBD -TAC versus CD3/CD28 -CBD -TAC.
Supplemental Table 6. Dual-Contrast CBD Modulation Analysis in Effector-Memory T (TEM).
Supplemental Table 7. Pathway Analysis with KEGG for Effector-Memory T (TEM) cells.
Supplemental Table 8. Cell-Cell Communication Result Across Level 2 Annotation.
Supplemental Table 9. Network of CBD-Regulated Pathways in Effector-Memory T (TEM). Cells.
Supplemental Table 10. Dual-Contrast CBD Modulation Analysis in B CD70hi Cells.
Supplemental Table 11. Pathway Analysis with KEGG for B CD70hi Cells.
Supplemental Table 12 | Dual-Contrast CBD Modulation Analysis in Proliferating T cells.
Supplemental Table 13 | Pathway Analysis with KEGG for Proliferating T Cells.
Supplemental Table 14 | Differentially Expressed Genes (DEGs) Across the Three Annotation Levels.
Supplemental Table 15 | Cell Proportion Forest Plot with Tacrolimus.
Supplemental Table 16 | Cytokine Data.
Supplemental Table 17 | Flow Cytometry Cell Counts and Proportions.
